# Disabling the Entatic Control of Methionine Ligation through Additive Destabilization of Ferric Cytochrome *c*

**DOI:** 10.1021/acs.inorgchem.5c00839

**Published:** 2025-06-11

**Authors:** Morgan E. Reik, Taylor C. Rickett, Kevin R. Hoke, Ekaterina V. Pletneva

**Affiliations:** † Department of Chemistry, 3728 Dartmouth College, Hanover, New Hampshire 03755, United States; ‡ Department of Chemistry and Biochemistry, 5659 Berry College, Mount Berry, Georgia 30149, United States

## Abstract

The entatic state plays a key role in modulating the functional properties of metal sites, especially in proteins. In cytochrome (cyt) *c*, a hydrogen-bonding network contributes to stabilization of Met80 ligation to the heme iron, supporting the dual functions of this metalloprotein as an electron carrier in respiration and a peroxidase in apoptosis. We have prepared a cyt *c* variant in which both Thr49 and Thr78 within this network have been replaced with Val residues. Spectroscopic and electrochemical experiments suggest that the ferric form of the protein no longer has Met80 coordinating the heme iron, while the ferrous form preserves this interaction. Thermodynamic analyses demonstrate how perturbations at the periphery of the heme introduced by the mutations affect the stabilities of Met-, Lys-, and H_2_O-ligated conformers. The foldon structure enables the propagation of destabilization effects to the region implicated in the entatic control of the Met80 ligation. The extent of destabilization is similar for the ferric and ferrous Met-ligated conformers, but the ligation outcome differs because the global stability of the protein and stabilities of its foldons depend on the redox state of the heme iron. The stability of low-energy foldons could be tuned in other metalloproteins to engineer redox-linked switchable functions.

## Graphical abstract

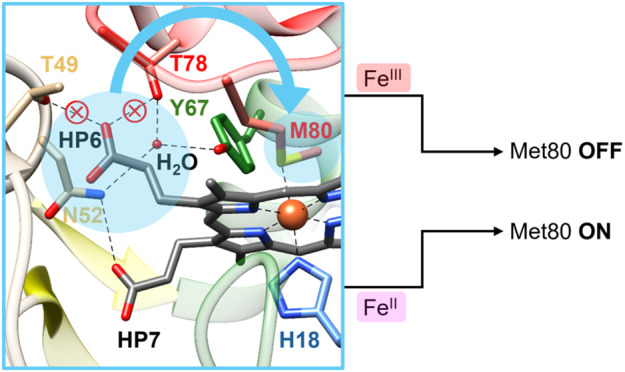

## Introduction

The entatic state,^1,2^ in which the surrounding protein frame strains the active site, is of key importance in bioinorganic chemistry. This state serves to minimize structural changes upon electron transfer (ET) and could also benefit catalysis in substrate binding and activation.^3–9^ The principles of the entatic state are not limited to metalloproteins, and there are examples of small complexes where strain can similarly impose a geometry on the metal sites to lower the activation barrier for chemical reactions.^2,10–12^ Both rigid frames and more flexible rack-induced structures have been introduced as models for this phenomenon.^1,2,6^ The latter is particularly relevant in metalloproteins, where structural elements beyond the active site must be taken into account. The energetics of intramolecular interactions and stability of conformers in the polypeptide ensemble are important to consider in understanding protein structure and function and connecting them to properties of the entatic state.

Herein, we have employed a small protein cytochrome (cyt) *c* to probe the stability of differently ligated heme species and the role of interactions at the periphery of the heme on the entatic control of its Met ligation. This protein has been an informative model for studies of ET and metalloprotein folding.^13–18^ The structure of cyt *c* is composed of units (“foldons”) that differ in stability and, in order from least to most stable, are known as the infrared (residues 40–57), red (residues 70–85), yellow (residues 37–39, 58–61 β-strands), green (residues 19–36 and 60s α-helix), and blue (N- and C-terminal α-helices) foldons (Figure 1A).^14,16–19^ The foldons, heme, and iron ligands are integrated into an elaborate intraprotein hydrogen-bonding network (Figure 1A). This network plays a role in preserving Met80 ligation to the heme iron in this electron-carrier protein and minimizing reorganization energy as the heme iron cycles between ferric and ferrous states. The ligation by a soft amino-acid ligand Met raises the potential of the heme iron, which is important for its role in electron delivery to terminal oxidases.^20^ Cyt *c* also acts as a peroxidase in the early steps of apoptosis, and to enable this function, Met80 has to dissociate from the heme iron.^13,21^ Multiple triggers cause Met80 displacement, including solution pH,^22^ binding of small molecules,^23–25^ and interactions of the protein with denaturants and membranes.^26,27^ Consistent with broad hard/soft-acid/base principles, the loss of the thioether Met ligation is particularly facile for ferric cyt *c*.^28,29^ The two redox functions of cyt *c* are thus ultimately linked to the Met80 ligand and entatic control of this ligation.

**Figure 1:**
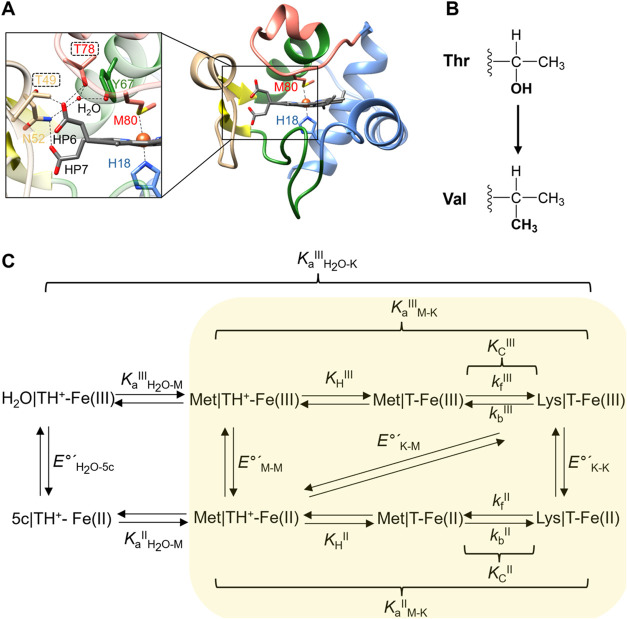
(A) Foldons in yeast *iso*-1 cyt *c* (PDB: 1YCC),^30^ color-coded according to the notation of Englander and co-workers (adapted with permission from ref 17, copyright 2006 Elsevier). The hydrogen-bonding network including the three residues implicated in the entatic control of the Met80 ligation to the heme iron and residues connecting to HP6 is also shown. (B) Mutations Thr-to-Val examined in this work. (C) Scheme depicting the states associated with the pH-dependent transitions in cyt *c* and deprotonation of a trigger group. “T” is a state in which the moiety triggering the ligand-switching reaction is deprotonated, whereas “TH^+^” is a state in which this group is protonated. A part of the scheme associated with the alkaline transition is highlighted. In ferric yeast *iso*-1 cyt *c*, the p*K*_aM‑K_^III^ value is ∼8.5 (tabulated in ref 31, copyright 1989 American Chemical Society).

Studies by Solomon et al. have revealed that the entatic stabilization of the Met ligation in ferrous cyt *c* is relatively modest (∼17 kJ mol^–1^) and suggested a role of Asn52, Tyr67, and Thr78 in this stabilization (Figure 1A).^32,33^ These residues are connected by hydrogen bonds and located right next to Met80; here, we refer to them as the entatic triad. Applying perturbations to the triad is an obvious choice to disable the entatic control and, indeed, mutations of these three residues have been reported to affect ligation in cyt *c*, favoring coordination of Lys73 and Lys79 instead of the native Met80.^34–37^ The Met-to-Lys ligand switch in cyt *c*, known as the alkaline transition, serves as a convenient proxy for the propensity of Met80 for coordination to the heme iron.

We wondered whether the region of entatic control extends beyond the triad and whether there may be energetic differences in disabling this control in ferric and ferrous forms of cyt *c*. Residue Thr78 of the entatic triad, as well as another Thr, Thr49, are connected to the intraprotein hydrogen-bonding network through a heme propionate HP6. Yeast *iso*-1 cyt *c* is one of less stable cyt proteins and we have previously reported that, in comparison to a parent yeast *iso*-1 cyt *c* variant K79G, individually mutating these two Thr residues to Val (Figure 1B) destabilizes the Met80-ligated state in the ferric protein by 9.7 ± 3.5 and 7.7 ± 3.5 kJ mol^–1^ for the yeast *iso*-1 cyt *c* variants T49V/K79G and T78V/K79G, respectively.^35^ While the p*K*_aM‑K_^III^ value of the Met-to-Lys transition (Figure 1C) was shifted to 6.7 in T49V/K79G and T78V/K79G, the ferric Met conformer was still observable in both variants. We hypothesized that perturbations upon the two Thr-to-Val mutations will further destabilize the protein and may translate through its foldon structure toward the heme iron to disable the entatic control of the Met80 ligation, yielding a conformer that does not coordinate Met despite having this residue near the heme iron. A conformation of cyt *c* in a Met-off state has previously been observed in a crystal structure,^23^ but in solution, the protein remained Met-ligated.^38^

In the variant T49V/T78V/K79G of yeast *iso*-1 cyt *c* that we describe in this study, the Met-ligated conformer is no longer populated at equilibrium for the ferric protein in solution but is observed in the ferrous protein. By quantifying the Gibbs free energies of both observable and nonobservable states, we rationalize redox-dependent changes in Met ligation and the implications for the disruption of the entatic state. Further, we use a free-energy framework to elucidate the structural effects of these replacements in the Met-, Lys-, and H_2_O-ligated cyt *c* conformers. Because functions of many metalloproteins rely on ligand dissociation or substitution, and this change in ligation ultimately relates to thermodynamic stability of differently ligated conformers, insights from this work may help in the design of metalloproteins that switch their structure and reactivity upon a redox input.

## Materials and Methods

### General

Water was purified to a resistivity of 18.2 MΩ cm using a Barnstead E-Pure Ultrapure Water Purification System. Reagent-grade chemicals were used to prepare all of the solutions. Fitting of spectral data was performed using MATLAB R2024b, and NMR data were processed with MestReNova 14.1.1.^39^ Protein models were visualized using UCSF Chimera 1.16.^40^

### Site-Directed Mutagenesis, Protein Expression, and Purification

The pRbs_BTR1 plasmid encoding yeast *iso*-1 cyt *c* was used for subsequent site-directed mutagenesis.^41^ This parent plasmid contained K72A and C102S mutations to prevent coordination of Lys72 of yeast *iso*-1 cyt *c* to the heme iron^42^ and disulfide bond formation, respectively. Additional mutations were introduced using a QuikChange kit (Agilent), and resulting plasmids were sequenced using the Dartmouth College Genomics and Molecular Biology Shared Resources. Protein expression and purification were performed as previously described.^43^

### Spectroscopic Measurements

Agilent 8453 or Shimadzu UV-1201 spectrophotometers were used to record electronic absorption spectra. Protein concentrations were quantified using the Soret band with extinction coefficients determined from hemochrome assays.^44^ Ferric and ferrous forms of the proteins were obtained as previously described,^34^ by oxidation with potassium ferricyanide (K_3_Fe­(CN)_6_, Acros Organics) and reduction with sodium dithionite (Na_2_S_2_O_4_, Fisher Chemical), respectively.

Circular dichroism (CD) spectroscopic experiments were performed on a JASCO-J815 CD spectrometer. Data were collected in the near-UV (250–300 nm) and far-UV (200–250 nm) regions to probe protein tertiary and secondary structure, respectively. Preparation of protein samples and cuvettes for these measurements was as previously described.^35^ Fluorescence spectroscopy measurements were conducted on a Jobin Yvon Spectrolog III fluorimeter (Horiba Scientific). The following parameters were chosen to probe Trp59 fluorescence: λ_ex_ = 290 nm and λ_em_ = 300–400 nm, with emission and excitation slit widths of 10 nm. *N*-Acetyl-tryptophanamide (NATA, Sigma Life Science) was used as a reference for the unquenched Trp.

Electron paramagnetic resonance (EPR) spectra were collected at 10 K on a Bruker EMX X-band EPR. Ferric samples were prepared by exchanging the proteins into 100 mM sodium phosphate buffer at pH 7.4 and 15% v/v glycerol. Protein concentrations were 0.75–1 mM. Measurements were done using 16 scans and a sweep width of 4400 G. All other parameters were as previously described.^38^

^1^H NMR spectra were collected on a 500 MHz Bruker NMR spectrometer as previously described.^34^ Ferric protein samples were prepared in 50 mM sodium phosphate buffer at pD 7.4 in 100% D_2_O (Cambridge Isotope Laboratories). Ferrous protein samples were prepared in a degassed buffer containing 50 mM sodium acetate (pH 4.5), 50 mM sodium phosphate (pH 7.4), 50 mM sodium borate (pH 9.0–10.0), or 50 mM *N*-cyclohexyl-3-aminopropanesulfonic acid (CAPS) (pH 10.25–10.75) under a nitrogen atmosphere; the final samples contained 10% D_2_O.

### pH Titration Experiments

The pH of all solutions was recorded by using an AB15 pH meter (Fisher Scientific). pH titrations (pH 2–11) monitoring the Soret (300–650 nm) and charge-transfer (600–750 nm) regions of the electronic absorption spectra of ferric T49V/T78V/K79G were performed as previously described.^35^ Singular value decomposition (SVD) analyses on the spectral data of ferric T49V/T78V/K79G from pH titrations were performed as previously described.^35^ Global fitting of the most weighted V-vectors from the ferric titration over pH 2–11 to eq 1 was done in MATLAB R2024b, where *t_i_*, *n_i_*, and p*K*_app,*i*_ are the amplitude, slope, and apparent p*K*_a_ value of transition *i*, respectively, and *C* is the *y*-intercept.

1 $$f\left(x\right)=\sum_{i}\frac{t_{i}}{1+10^{n_{i}\left(pK_{\mathrm{app},i}-\mathrm{pH}\right)}}+C$$

As T49V/T78V/K79G became readily oxidized, a full pH titration experiment was difficult to perform with the ferrous protein. Instead, individual samples of ferrous T49V/T78V/K79G were prepared in a glovebox in degassed buffers of varying pH values over the range of 7.4–12.9. Protein samples were reduced with 10 mM Na_2_S_2_O_4_ and subsequently run on a PD-10 column to remove excess reducing agent. Upon determining the protein concentration by electronic absorption spectroscopy, 20–30 μL of the concentrated protein stock was added to degassed 20 mM sodium phosphate (pH 7.4–8, pH 11–13) or 20 mM borate (pH 9.5–10) buffers inside the glovebox to create samples at a concentration of 5–7 μM. Final protein samples contained 0.5 mM Na_2_S_2_O_4_. Signals were recorded by using electronic absorption spectroscopy at 415 nm with a path length of 1 cm and CD spectroscopy at 222 nm using a path length of 4 mm. Samples were prepared in quartz cuvettes with a threaded top and sealed with a septum seal prior to data collection. The dependence of absorbance *A* on pH was fit to eq 2, where *A*_HA_ is the absorbance of the protonated species and *A*_A‑_ is the absorbance of the deprotonated species.

2 $$\mathrm{pH}-pK_{a}=\log\left(\frac{A-A_{\mathrm{HA}}}{A_{A-}-A}\right)$$

### Direct Electrochemistry

Gold working electrodes were surface-modified using 3-mercaptopropanol.^35^ Samples of T49V/T78V/K79G in 100 mM sodium acetate at pH 5.0 or 100 mM sodium phosphate at pH 6.0 or 7.4 buffer were examined at 21 ± 1 °C by cyclic voltammetry (CV) using a saturated calomel reference electrode (SCE) and platinum wire counter electrode. The reference electrode was separated from the others by a Luggin capillary. Both electrochemical cell compartments were first filled with buffer and vigorously sparged with argon before the working cell contents were replaced by the sample, which was then allowed to equilibrate under an argon flow for 30–40 min before introduction of the working electrode. Ultrahigh-purity argon was passed through an oxygen scrubbing catalyst column en route to the cell. CV was carried out using a CH Instruments 630F potentiostat from 0.1 to 20 V/s, with ten or more intervening scan rates. Preconditioning potential holds were used before each run to ensure an oxidized sample at the electrode. Reduction potentials were corrected to the standard hydrogen electrode (SHE) using *E*_SHE_ = *E*_SCE_ + 244 mV.^45^ Subtraction of background currents was done using Qsoas.^46^ Digital simulations of CV were carried out as described previously.^35^

### Spectroelectrochemistry

Spectroelectrochemical titrations of T49V/K79G, T78V/K79G, and T49V/T78V/K79G were performed in both oxidative and reductive directions using a three-electrode system (Pine Research) consisting of a honeycomb working electrode, a platinum auxiliary electrode, and an Ag/AgCl gel reference electrode under the control of a WaveNow USB potentiostat.^47^ Solutions of 100 μM proteins were prepared in a 100 mM phosphate buffer at pH 7.4 containing the following mediators: 25 μM *p*-benzoquinone, 25 μM *N*,*N*,*N*′,*N*′-tetramethyl-*p*-phenylenediamine (TMPD), and 25 μM 1,2-naphthoquinone. At each applied potential, an electronic absorption spectrum was collected in the range from 500 to 600 nm after equilibration for 20 min. The fraction of ferrous protein *X*_Fe(II)_ was determined from absorbance readings at 550 nm using eq 3. Baseline correction was performed as previously described.^48^

3 $$X_{\mathrm{Fe}\left(\mathrm{II}\right)}=\frac{A_{550,\mathrm{sample}}-A_{550,\mathrm{Fe}\left(\mathrm{III}\right)}}{A_{550,\mathrm{Fe}\left(\mathrm{II}\right)}-A_{550,\mathrm{Fe}\left(\mathrm{III}\right)}}$$

The dependencies of *X*_Fe(II)_ on the applied potential were fit to eq 4, where *E*_m_ is the midpoint potential and *n* is the number of electrons transferred.

4 $$X_{\mathrm{Fe}\left(\mathrm{II}\right)}=\frac{1}{1+\exp\left[\frac{\left({E-E}_{m}\right)\times n\times F}{\mathrm{RT}}\right]}$$

### Kinetics Measurements

Kinetics measurements were performed using a Biologic SFM-300 stopped-flow instrument.^47^ A flow rate of 6 mL s^–1^ was used, yielding a mixing dead time of 7.5 ms. The formation of the Met-ligated ferric species was evaluated using a previously described oxidation experiment.^49^ Ferrous T49V/T78V/K79G (0.3 mM) was mixed with 1.8 mM K_3_Fe­(CN)_6_ in a 10 mM sodium phosphate buffer at pH 7.4 containing 100 mM NaCl to yield final concentrations of the protein and K_3_Fe­(CN)_6_ of 50 μM and 0.3–1.5 mM, respectively. Changes in the absorbance at 695 nm, indicative of Met ligation to the heme iron, and at 620 nm, indicative of H_2_O ligation,^50^ were examined. Kinetics at 620 nm were fit to a biexponential decay.

Kinetics of Lys dissociation from the ferric heme iron in T49V/T78V/K79G were evaluated by monitoring changes in the Soret absorption band upon imidazole (Im) binding to the heme iron. Stock solutions of 50 μM protein and 150 mM Im were prepared in 100 mM sodium phosphate buffer at pH 7.4 and yielded final concentrations of ∼8 μM protein and 25–125 mM of Im. The reaction progress was monitored for 60 s, and changes in absorbance at 398 nm were fitted to monoexponential decay functions to yield *k*_obs_^Im^. Rate constants *k*_off_^Lys^ and *k*_off_^Im^, corresponding to dissociation from the ferric heme iron of Lys and Im, respectively, were determined from fits to eq 5. The pH-independent ligand-dissociation equilibrium constant *K*_D_ in eq 5 was obtained from titration experiments.^34^

5 1kobsIm=1koffLys+KDkoffIm[Im]

### Denaturation Experiments

The global stability of ferric T49V/T78V/K79G was assessed from denaturation experiments using guanidine hydrochloride (GuHCl) (Ultrapure, 99%, Thermo Scientific). Experimental parameters and details of protein solution preparation in GuHCl at pH 4.5 and pH 7.4 were as previously described.^35^ The dependencies of CD signals at 222 nm on the concentration of GuHCl were fit to eq 6,^51^ where *m*_f_ and *m*_u_ are the slopes of the lines that describe trends in the signals for the folded and unfolded states, respectively, *b*_f_ and *b*_u_ are the corresponding intercepts, *m*_D_ is the slope of the transition, and *C*_m_ is the concentration of GuHCl at the midpoint of the transition.

6 $$f\left(x\right)=\frac{m_{f}\left[\mathrm{GuHCl}\right]+b_{f}+\left(m_{u}\left[\mathrm{GuHCl}\right]+b_{u}\right)\;\exp\left[\frac{m_{D}\left(\left[\mathrm{GuHCl}\right]-C_{m}\right)}{\mathrm{RT}}\right]}{1+\exp\left[\frac{m_{D}\left(\left[\mathrm{GuHCl}\right]-C_{m}\right)}{\mathrm{RT}}\right]}$$

## Results

### The Protein Fold and Axial Ligation in T49V/T78V/K79G

The structural consequences of combining T49V and T78V mutations in *iso*-1 cyt *c* were examined by probing the global fold and axial ligation in the variant. The far-UV CD spectra of T49V/T78V/K79G in its ferric (Figure S1A) and ferrous (Figure S1B) states exhibit only minor differences compared to those of other variants, suggesting little (or no) changes in the protein secondary structure. Changes in the tertiary structure are more pronounced, as evidenced by the signal decrease in the near-UV CD spectra (Figure S1C,D). The minima in the near-UV CD spectra at 282 and 288 nm of ferric cyt *c* have been previously assigned to Trp59.^52,53^ The increase in Trp59 fluorescence in T49V/K79G and T49V/T78V/K79G (Figure S2A,B) suggests a greater Trp59-to-heme distance relative to those in other variants. We have previously commented on the structural rearrangements in T49V/K79G;^35^ these effects are now enhanced in T49V/T78V/K79G (Figure S2A,B). However, the increase in the intensity of the Trp59 signal is only a fraction (∼25%) of that seen upon denaturation of the protein. Collectively, the CD and Trp59 fluorescence findings suggest that at pH 7.4, the secondary and much of the tertiary structure are preserved upon Thr-to-Val substitutions to create the three variants we compare in this work.

The position of the Soret band (Figure 2A) in the electronic absorption spectrum of ferric T49V/T78V/K79G at pH 7.4 is similar to that in the Lys-ligated ferric T49V/K79G and T78V/K79G, but blue-shifted compared to that in the Met-ligated K79G.^35^ A 695 nm charge-transfer band, characteristic of Met ligation,^50^ is lacking in the spectrum of T49V/T78V/K79G (Figure 2B). Instead, a small 620 nm charge-transfer band is present, suggesting a population of the H_2_O-ligated conformer under these conditions.^50^ The *g*-values in the EPR spectra are distinct from those of the Met-ligated K79G but are comparable to those of the Lys-ligated T49V/K79G and T78V/K79G (Figure 2C) as well as other Lys-ligated cyt *c* proteins.^54^ In addition to the low-spin signal in the EPR spectrum of T49V/T78V/K79G, a small population of high-spin species is also apparent (Figure 2C). The positions of the heme methyl signals in the ^1^H NMR spectra of T49V/T78V/K79G (Figure 2D) are also consistent with Lys ligation.

**Figure 2:**
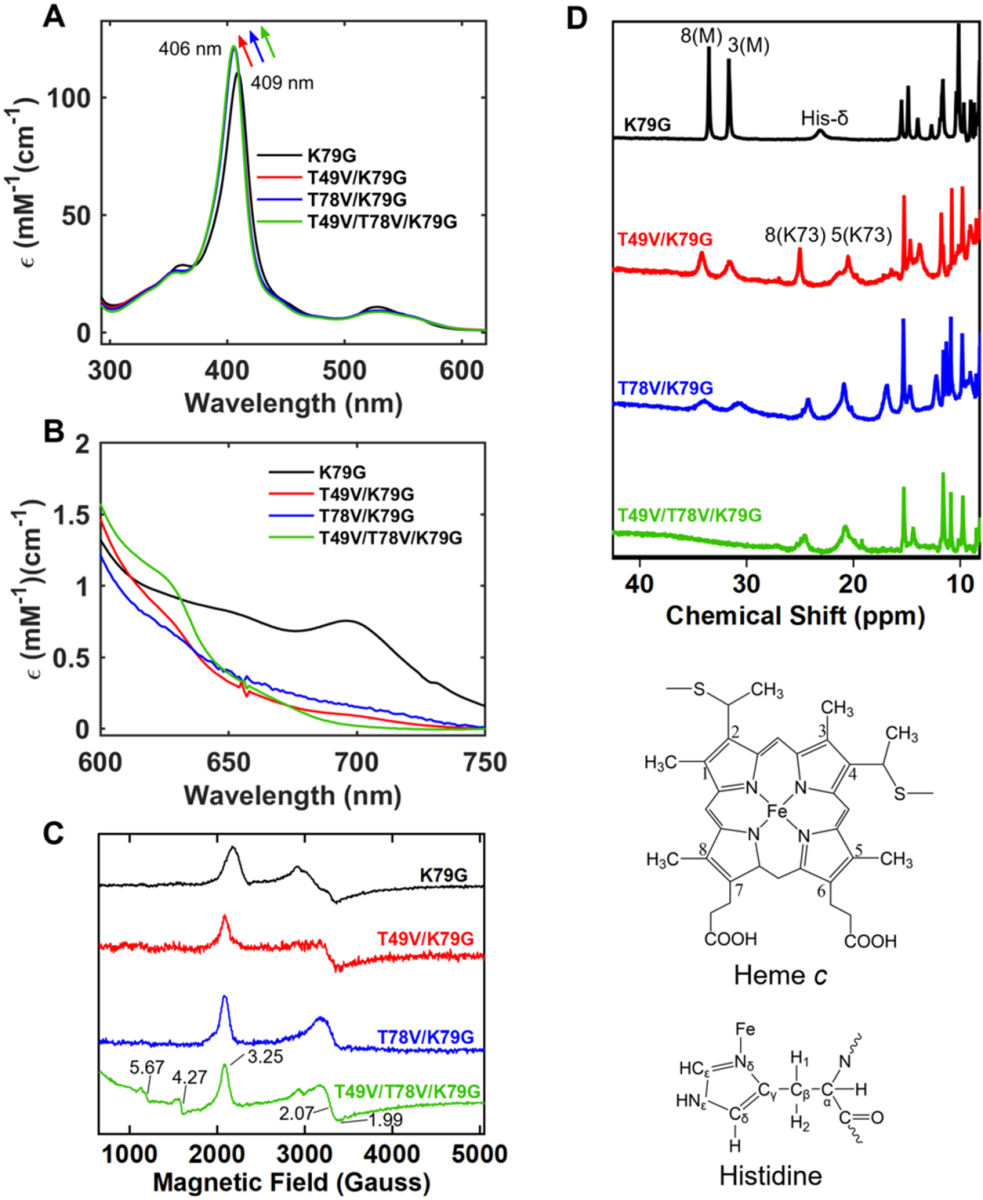
Spectra of ferric variants at pH 7.4. Electronic absorption monitoring of (A) the Soret and (B) the charge-transfer bands. (C) EPR (at 10 K). (D) NMR and structures of heme *c* and the His side chain depicting labeling nomenclature.

At pH 4.5, the blue-shifted Soret band (Figure S3A), the lack of the 695 nm charge-transfer band (Figure S3B) in the electronic absorption spectra as well as the absence of the ^1^H NMR signals at frequencies associated with methyls of the Met-ligated heme (Figure S4) all suggest that ferric T49V/T78V/K79G does not contain a detectable (or any) Met-ligated species. The intensity of the 620 nm charge-transfer band is greater than that at pH 7.4, suggesting an increase in the population of the H_2_O-ligated species at the lower pH.^50^ While some perturbations in the tertiary structure of the protein do take place, the protein is largely folded at pH 4.5 (Figure S5).

To assess whether species other than the Lys- and H_2_O-ligated ones are involved in the pH-dependent equilibria, a titration of ferric T49V/T78V/K79G in a wide pH range, from 2 to 11, was performed (Figure S3A). The 695 nm charge-transfer band was not detectable throughout this entire pH range (Figure S3B). SVD analyses suggested that three states (and two transitions) were necessary to describe spectral changes as a function of pH. Global fitting of the most weighted V-vectors from titrations monitoring the Soret and charge-transfer regions (Figure S3C) to eq 1 revealed p*K*_a_^III^ values of the two transitions and the number of associated protons *n* (Table 1). The position of the Soret band at higher pH (∼6.5–9.4) (Figure S3A) matches that of the Lys-ligated species at pH 7.4 (Figure 2A). Spectral characteristics of the high-spin ferric heme iron species, such as the 620 nm charge-transfer band, are evident at lower pH (∼2.0–6.5). Thus, we attributed the p*K*_a_^III^ = 6.5 ± 0.1 to the transition between the H_2_O- and Lys-ligated species (Figure 3A,B and Table 1). This p*K*_a_^III^ value is lower than the p*K*_aM‑K_^III^ = 8.6 ± 0.1 for K79G (Table 1).^38^ Only one transition is resolved at pH < 6.5 in T49V/T78V/K79G, in contrast to two transitions for T49V/K79G and T78V/K79G.^35^ K79G similarly has one acidic transition with a p*K*_a_ value of 3.0 ± 0.1,^38^ which has been attributed to protonation of His26 and His18.^55^ The p*K*_a_^III^ value of this (acidic) transition for T49V/T78V/K79G is 2.4 ± 0.1 (Table 1).

**Table 1:** Results from Global Fitting of V-Vectors from SVD Analysis of Ferric Variants of Yeast *iso*-1 Cyt *c*

variant	p*K*_a_^III^	*n* ^a^	transition
K79G	3.0 ± 0.1^b^	2.3 ± 0.3	H_2_O/H_2_O → His/Met
	8.6 ± 0.1^b^	0.9 ± 0.3	His/Met → His/Lys
	12.5 ± 0.1^c^	0.8 ± 0.2	His/Lys → His/OH^–^
T49V/K79G^d^	2.6 ± 0.1	1.6 ± 0.1	H_2_O/H_2_O → His/H_2_O
	3.4 ± 0.1	1.5 ± 0.1	His/H_2_O → His/Met
	6.7 ± 0.1	0.9 ± 0.1	His/Met → His/Lys
T78V/K79G^d^	2.9 ± 0.1	1.6 ± 0.1	H_2_O/H_2_O → His/H_2_O
	5.0 ± 0.1	1.5 ± 0.1	His/H_2_O → His/Met
	6.7 ± 0.1	0.9 ± 0.1	His/Met → His/Lys
T49V/T78V/K79G	2.4 ± 0.1	2.0 ± 0.1	H_2_O/H_2_O → His/H_2_O
	6.5 ± 0.1	0.9 ± 0.1	His/H_2_O → His/Lys

a Number of protons from the fit to eq 1.

b Reported in ref 38.

c Reported in ref 56.

d Reported in ref 35.

**Figure 3:**
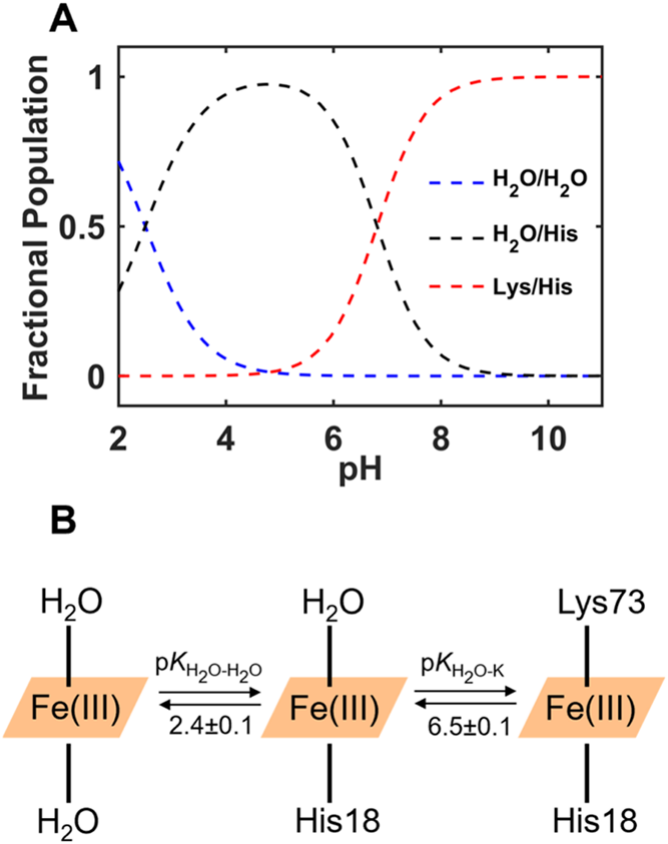
(A) Fractional populations of the species and (B) inferred transitions from the pH titration of ferric T49V/T78V/K79G. Corresponding spectra are shown in Figure S3.

The Soret band of ferrous T49V/T78V/K79G at pH 7.4 is at the same position as in other 6-coordinate low-spin cyt *c* variants (Figure 4A).^35^ The Soret band of this variant has λ_max_ = 415 nm within the pH range from 7.4 to 13, but its molar absorptivity changes (Figure S6A); the fit of the dependence to eq 2 has yielded a p*K*_a_^II^ value of 11.0 ± 0.2 (Table 2). The upfield signals in the ^1^H NMR spectra of ferrous T49V/T78V/K79G (Figures 4B,C and S6B) suggest that in the pH range between 4.5 and 10.8, the heme iron is Met-ligated. These signals return after subsequent oxidation and rereduction of the heme iron in this variant, suggesting reversibility of the redox-linked ligand exchange. At pH values near pH 11, the α-helical signal in the CD spectra of the ferrous protein is smaller in magnitude; this signature fully disappears by pH 12.7 (Figure S6C), suggesting that p*K*_a_^II^ may correspond to or coincide with protein denaturation. Collectively, these findings imply that Met80 persists as the ligand to the ferrous heme iron until this variant denatures and that this form is accessible through the reduction of the Lys-ligated ferric protein.

**Table 2:** Parameters for the Alkaline Transition of Ferrous Variants of Yeast *iso*-1 Cyt c

variant	*k*_f_^II^ (s^–1^)	*k*_b_^II^ (s^–1^)	p*K*_a_^II^	p*K*_a_^II^ ^a^
K79G	(2.8 ± 2.0) × 10^–3^ ^b^	3.5 ± 0.5^b^	15.6 ± 0.7^b^	na^c^
T49V/K79G	(1.4 ± 1.0) × 10^–1^ ^b^	7.0 ± 0.5^b^	12.5 ± 0.4^b^	12.3 ± 0.2
T78V/K79G	(2.3 ± 1.7) × 10^–3^ ^b^	11.5 ± 0.5^b^	13.7 ± 0.7^b^	13.2 ± 0.1
T49V/T78V/K79G	nd^d^	16 ± 1.0^e^	11.0 ± 0.2^f^	11.3 ± 0.1

a Calculated using exp­[(*nFE*_K‑M_°′ *–nFE*_K‑K_°′)/(−*RT*)] = *K*_eq_^II^ at pH 7.4, where *K*_eq_ = 10^pH‑p*K*a^.

b Reported in ref 35.

c K79G does not undergo Met-to-Lys ligand-switching at pH 7.4, and thus a cross-potential cannot be used to calculate a theoretical p*K*_a_^II^ value.

d As T49V/T78V/K79G was readily oxidized, preventing the obtainment of a full ferrous pH titration, decoupling *K*_a_^II^ into *K*_C_^II^ and *K*_H_^II^ and thus calculating *k*_f_^II^ proved challenging for T49V/T78V/K79G.

e Obtained from CV using digital simulation.

f Determined from linear fit to the pH titration of ferrous protein.

**Figure 4:**
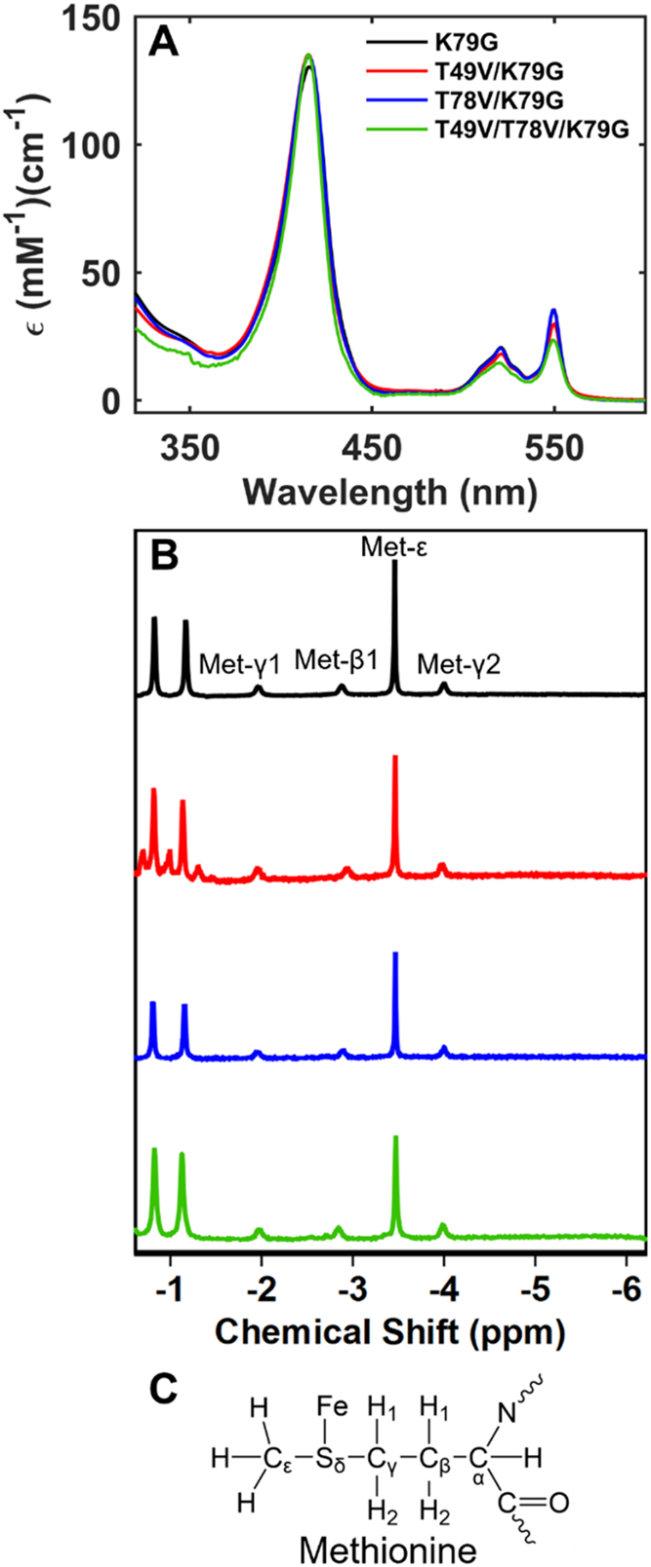
(A) Electronic absorption and (B) NMR spectra of ferrous variants at pH 7.4. (C) Structure of the Met side chain depicting labeling nomenclature.

### Direct Electrochemistry of T49V/T78V/K79G

Cyclic voltammetry (CV) was used to determine reduction potentials of the differently ligated forms of T49V/T78V/K79G and to evaluate redox-linked ligand switching. Starting at the upper potential limit, scanning to below −100 mV yields a distinct peak for the reduction of ferric T49V/T78V/K79G at pH 7.4 (Figures 5 and S7). Spectroscopic studies were performed at equilibrium (Figure S3A,B), suggesting that under these conditions, the ferric protein is predominantly Lys-ligated (Figure 3A). The amount of the H_2_O-ligated species (7–12%, Figure 3A and Table 3) provides a small contribution to the current response, as seen in scans over a narrower, higher potential range (Figure S8A). Redox-linked ligand switches may yield CV responses that depend on the scan rate.^29^ As was the case for T49V/K79G and T78V/K79G, a peak due to the reoxidation of the ferrous Lys-ligated species is not apparent at slower scan rates (Figure 5A) but grows in intensity as the scan rate is increased (Figure 5B). The pair of peaks for this species correspond to a potential of −137 ± 4 mV vs SHE (at a scan rate of 20 V/s). The value is consistent with the potentials of the Lys-ligated cyt *c* conformers.^28,57,58^

**Table 3:** Thermodynamic Parameters for Ferric Variants of Yeast *iso*-1 Cyt *c* from GuHCl Unfolding Measurements at pH 7.4

variant	axial ligand^a^	*m*_D_^III^(kJ mol^–1^ M^–1^)^b^	Δ*G*_u_^III°′^ (kJ mol^–1^)^b^
K79G^c^	Met80 (100%)	16.9 ± 2.8	22.1 ± 3.0
T49V/K79G^c^	Lys73 (78–85%), Met80 (15–22%)	14.9 ± 3.0	16.4 ± 3.5
T78V/K79G^c^	Lys73 (78–85%), Met80 (15–22%)	15.5 ± 2.8	19.1 ± 3.4
T49V/T78V/K79G^c^	Lys73 (88–93%), H_2_O (7–12%)	13.7 ± 2.6	17.3 ± 3.3

a Calculated using the corresponding p*K*_a_^III^ value from the pH titration; ranges provided are determined from the error bars in the p*K*_a_^III^ values.

b Experimentally determined from GuHCl unfolding measurements (Figure S8B).

c Reported in ref 35.

**Figure 5:**
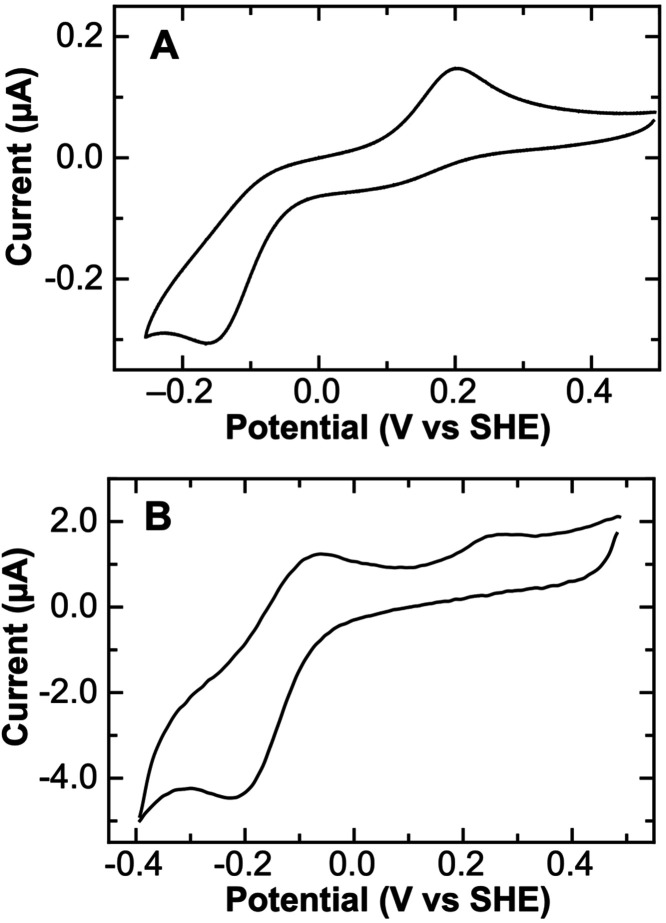
Cyclic voltammograms of T49V/T78V/K79G at (A) 0.1 and (B) 20 V/s at pH 7.4. Data shown are the second cycles, with background current subtracted. Noise reduction by Fourier filtering was applied to the 20 V/s data. The protein concentration was 135 μM.

In CVs of T49V/K79G and T78V/K79G, the oxidation and reduction gave pairs of well-defined peaks (at both low and high potentials), allowing for unambiguous determination of potentials of the two species.^29^ For T49V/T78V/K79G at pH 7.4, a peak at +200 mV (at a scan rate of 0.1 V/s) was observed on the return sweep to higher potentials (Figures 5 and S7A). Importantly, a prior scan to lower potential (to reduce the low-potential species first) was essential for the appearance of this signal (Figure S8B). A distinct voltammetric peak for the reduction of the higher-potential species, however, was not observed even at faster scan rates. Further, the current response for its reduction on the second cycle was difficult to distinguish from the sigmoidal current decay observed as the potential drops below that required for oxidation of its ferrous counterpart. As scan rates were increased to 20 V/s or higher, rapid reoxidation of the lower potential species competes with ligand exchange, diminishing the conversion to the higher-potential species and hence the magnitude of its signal in the CV (Figure 5B). Without clear peaks for both reduction and oxidation of the higher-potential species, accurate determination of its potential is challenging.

Lowering the pH diminishes the population of the Lys-ligated species due to shifts in equilibrium (Figure 3A). At pH 6.0, the intensity of the CV signal for the low-potential species (at −137 mV at pH 7.4) decreased; it fully disappeared at pH 5.0. The signal for the higher-potential species exhibited distinct peaks for both its reduction and oxidation at these lower pH values (Figure S9A,B), which allowed for the determination of the corresponding potential (150 mV and 175 mV at pH 6.0 and 5.0, respectively).^59^ The increase in the reduction potential upon lowering pH contrasts with the decrease in potential observed as the native cyt *c* undergoes an acidic transition in which its Met ligand is replaced by H_2_O.^60,61^ This finding, together with results from pH titrations at equilibrium (Figure S3A,B), argues against the higher-potential species at pH 7.4 being Met-ligated. Instead, the ligand to the heme iron in this species is most likely H_2_O. Examples of ferric H_2_O-ligated cyt *c* species having similar potentials include a transiently generated species for T78V/K79G at lower pH,^35^ a Phe82Trp variant,^62^ and wild-type cyt *c* absorbed directly or covalently linked to electrode surfaces.^63,64^

CV at fast scan rates was used to evaluate the kinetics of ligand switching. The scan rates needed to detect a peak for oxidation of transiently generated ferrous Lys-ligated T49V/T78V/K79G (0.9–1.1 V/s, Figure S7B) were faster than those needed for T49V/K79G and T78V/K79G.^29^ Digital simulations of voltammetry gave an estimate of 16 ± 1 s^–1^ for the rate constant *k*_b_^II^, which is of the same order of magnitude as *k*_b_^II^ values for the other variants (Table 2). Notably, the simulations required *k*_f_^III^ > 250 s^–1^ to conform with the lack of a distinct, nonsigmoidal peak at high scan rates for the reduction of the ferric species transiently generated at high potential (Figure S10). This value is 2 orders of magnitude faster than *k*_f_^III^ values for the other variants (Table 4),^35^ for which the ferric Met-ligated species is readily observed by CV.

**Table 4:** Parameters for the Alkaline Transition of Ferric Variants of Yeast *iso*-1 Cyt *c*

experimental					calculated	
variant	*k*_f_^III^ (s^‑1^)	*k*_b_^III^ × 10^2^ (s^‑1^)	p*K*_a_^III^	*E*_M‑M_°′ (mV)^a^	p*K*_a_^III^	*E*_M‑M_°′ (mV)^b^
K79G	8.4 ± 1.9^a^	10.6 ± 3.4^a^	8.6 ± 0.1^c^	262 ± 3	8.6 ± 0.2^a^	na^d^
T49V/K79G	2.0 ± 0.1^a^	6.1 ± 0.6^a^	6.7 ± 0.1^a^	197 ± 4	6.7 ± 0.2^a^	204 ± 6
T78V/K79G	4.1 ± 0.4^a^	6.0 ± 1.6^a^	6.7 ± 0.1^a^	275 ± 3	6.6 ± 0.3^a^	251 ± 6
T49V/T78V/K79G	≥300^e^	11.3 ± 1.0^f^	6.5 ± 0.1	nd^g^	4.9 ± 0.2^h^	252± 12

a Direct electrochemistry values were collected at 21 ± 1 °C and reported for Met-ligated K79G, T49V/K79G, and T78V/K79G, and other values indicated, reported in ref 35.

b Calculated using (*nFE*_K‑M_^°′^ – *RT* ln *K*_eq_^III^)/(−*nF*)­at pH 7.4 using *E*_K‑M_°′ from oxidative titrations (Table S1).

c Reported in ref 38.

d K79G does not undergo Met-to-Lys ligand-switching at pH 7.4, and thus a cross-potential *E*_K‑M_^°′^ cannot be used to calculate a theoretical *E*_M‑M_^°′^ value.

e The lower limit estimation from the K_3_Fe­(CN)_6_ oxidation experiment; *k*_f_^III^ ≈ 300 s^–1^ was used in calculations of *K*_C_^III^.

f Determined as *k*_off_^Lys^, the rate of Lys dissociation from imidazole binding kinetic studies.

g A direct reduction potential for the Met-ligated species *E*_M‑M_°′ was not observed for T49V/T78V/K79G.

h Calculated from ferric *K*_C_^III^ and *K*_H_^III^ (Table S2).

### Spectroelectrochemical Characterization of T49V/K79G, T78V/K79G, and T49V/T78V/K79G

While the reduction potential of the Met-ligated state (*E*_M‑M_^°′^) was readily determined using direct electrochemistry for the other cyt *c* variants in Table 4,^35^ this was not the case for T49V/T78V/K79G. However, the effective reduction potential for the conversion of the ferric Lys-ligated to ferrous Met-ligated species (*E*_K‑M_^°′^) can also be utilized in a thermodynamic analysis; these values were obtained from spectroelectrochemistry (Figure 6A–C and Table S1). To validate this approach, the following controls were performed. For T49V/K79G and T78V/K79G, the availability of *E*_M‑M_^°′^ from direct electrochemistry enabled the quantification of *K*_aM‑K_^II^. The experimental *E*_K‑K_^°′^ and *E*_K‑M_^°′^ values were employed to calculate p*K*_aM‑K_^II^ values for T49V/K79G and T78V/K79G, which agree within 0.6 units of those previously determined from other methods (Table 2). Further, when the *E*_M‑M_^°′^ values for T49V/K79G and T78V/K79G were calculated using the p*K*_aM‑K_^III^ and *E*_K‑M_^°′^ values, the results agreed within 30 mV with the corresponding values from direct electrochemistry (Table 4).^35^

**Figure 6:**
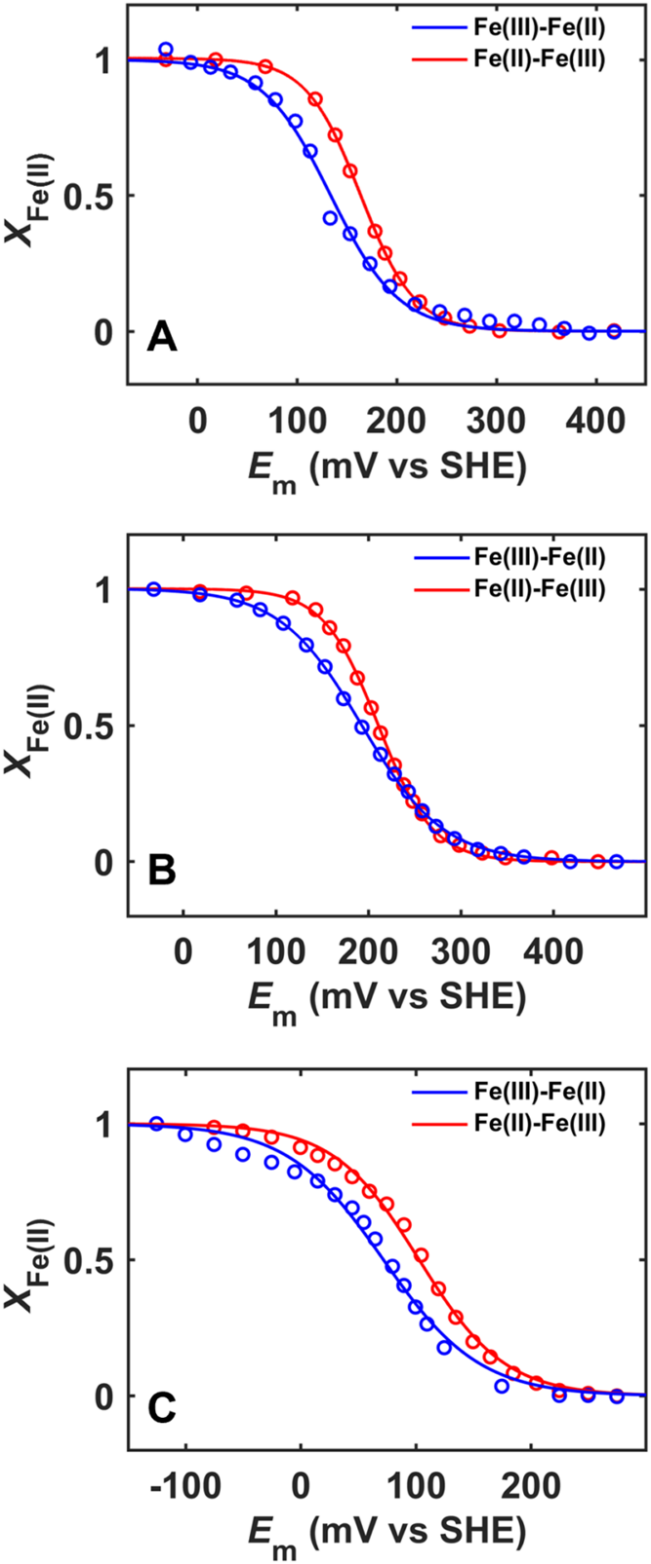
Spectroelectrochemical titrations of (A) T49V/K79G, (B) T78V/K79G, and (C) T49V/T78V/K79G at pH 7.4 and fits to eq 4. Results from fits of these data are listed in Table S1.

### Ligand-Switching Kinetics in Ferric T49V/T78V/K79G

The free energies of the ferric Met- and Lys-ligated states are related through *K*_C_^III^, which could be calculated using rate constants *k*_f_^III^ and *k*_b_^III^ (Figure 1C).^35,65,66^ To determine *k*_f_^III^ for T49V/T78V/K79G, as well as to assess whether Met ligation in ferric T49V/T78V/K79G could be observed transiently, kinetics of oxidation of the ferrous Met-ligated protein by K_3_Fe­(CN)_6_ were examined in a stopped-flow mixing experiment. Upon oxidation of the ferrous Met-ligated protein, the transiently generated ferric Met-ligated species are expected to undergo ligand switching to form the Lys-ligated species (Figure S11A).^49,67^ The 695 nm charge-transfer band was not observed at any point in the experiment, but a 620 nm charge-transfer band, indicative of H_2_O ligation, appeared immediately after mixing and its intensity decreased over time (Figure S11B,C). The major phase (>90%) of the 620 nm trace did not vary with K_3_Fe­(CN)_6_ concentration (Figure S11C). The lack of dependence on the concentration of K_3_Fe­(CN)_6_ suggests that bimolecular oxidation by this compound is fast, occurring during the dead time of the stopped-flow experiment. A decrease in the 620 nm absorbance after mixing, as well as the lack of the observable 695 nm band, suggests that the Met-ligated ferric transient is likely short-lived, and the observed trace describes the conversion of the H_2_O-ligated transient to the Lys-ligated product (Figure S11D). With the assumption that three half-lives are encompassed in the dead time of 7.5 ms, the rate constant for Met dissociation from the ferric heme iron could be set as ≥∼300 s^–1^ (Table 4). This lower bound is in agreement with the *k*_f_^III^ estimate from direct electrochemistry (Figure S10), and the value *k*_f_^III^ ≈ 300 s^–1^ was used in subsequent calculations of *K*_C_^III^.

Because the switch from Lys to Met is rate-limited by Lys dissociation from the heme iron in ferric cyt *c*,^68^ kinetics of Lys substitution by an exogenous ligand could provide information on the *k*_b_^III^ value. The *K*_D_ value of 380 ± 25 nM was obtained from analyses of changes at equilibrium in the electronic absorption spectra of T49V/T78V/K79G upon addition of Im (Figure 7A). Stopped-flow mixing experiments (Figure 7B) yielded a value of the rate constant *k*_off_^Lys^ = (11.3 ± 1.0) × 10^–2^ s^–1^. This value is of a similar order of magnitude as *k*_b_^III^ values for T49V/K79G and T78V/K79G determined from pH-jump and gated-ET experiments (Table 4).^35^

**Figure 7:**
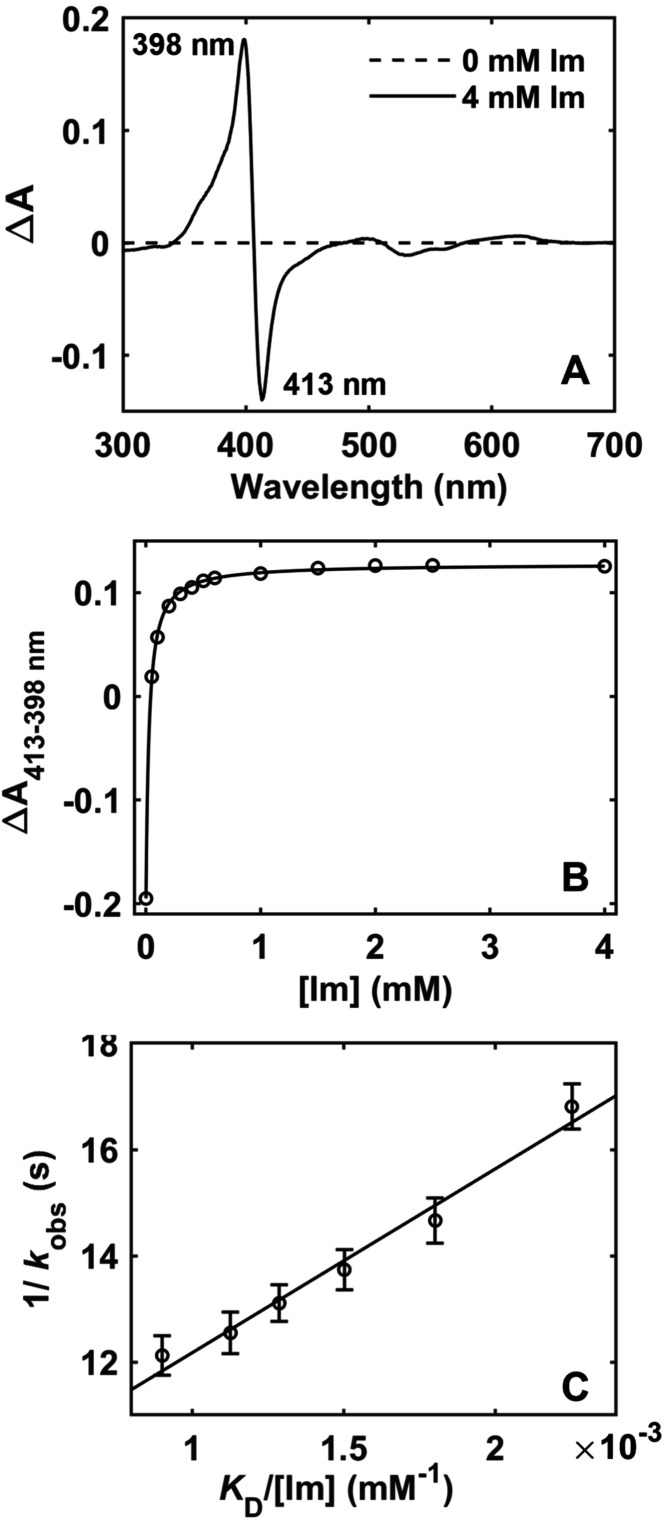
(A) Electronic absorption difference spectrum and (B) changes in the absorbance spectra upon addition of Im to ferric T49V/T78V/K79G (fit to eq 5, yielding *K*_D_ = (3.8 ± 0.25) × 10^–5^ M). (C) Linear fit of 1/*k*_obs_ vs *K*_D_/[Im] yielding *k*_off_^Lys^ = (11.3 ± 1.0) × 10^–2^ s^–1^.

### Global Stability of Ferric T49V/T78V/K79G

The Gibbs free energy of unfolding Δ*G*_u_^°′III^ from GuHCl denaturation experiments was used to quantify the global stability of the ferric Lys- and H_2_O-ligated states. At pH 7.4, where the majority of ferric T49V/T78V/K79G is Lys-ligated (Figure 3A and Table 3), the global stability of this variant is comparable to those of Lys-ligated T49V/K79G and T78V/K79G (Figure S12B and Table 3). At pH 4.5, where the majority of ferric T49V/T78V/K79G is H_2_O-ligated (Figure 3A and Table 6), the global stability of this variant remains comparable to that of T49V/K79G and T78V/K79G (Figure S12A and Table 6). For all four variants, the stability decreased upon lowering pH from 7.4 to 4.5.^35^ Meanwhile, denaturation of ferrous T49V/T78V/K79G presented challenges as the protein in the presence of the denaturant became readily oxidized during CD measurements. However, thermodynamic values from spectroelectrochemistry enabled estimation of the free energy of this ferrous protein. This approach was first validated with T49V/K79G and T78V/K79G (Tables 4 and 5).

**Table 5:** Reduction Potentials (*E* ^°′^) and Relevant Changes in Gibbs Free Energies for Variants of Yeast *iso*-1 Cyt *c* at pH 7.4

	reduction potential (mV vs SHE)				ΔΔ*G* (kJ mol^–1^)		
	experimental^a^		calculated^b^				
variant	*E*_K‑K_^°′^	*E*_K‑M_^°′^ ^c^	*E*_K‑M_^°′^ ^d^	*E*_K‑M_^°′^ ^e^	Δ*G*_M‑Fe(II)_^°′^ – Δ*G*_K‑Fe(III)_^°′^	Δ*G*_K‑Fe(II)_^°′^ – Δ*G*_K‑Fe(III)_^°′^	Δ*G*_M‑Fe(II)_– Δ*G*_M‑Fe(III)_^°′^
K79G	–145 ± 10^f^	nd^g^	332 ± 12	335 ± 42	–32.4 ± 3.9	14.0 ± 1.0^f^	–25.3 ± 0.3^f^
T49V/K79G	–141 ± 10^f^	147 ± 3	156 ± 7	158 ± 25	–15.5 ± 2.9	13.6 ± 1.0^f^	–19.0 ± 0.4^f^
T78V/K79G	–140 ± 4^f^	202 ± 1	234 ± 7	229 ± 40	–22.4 ± 4.5	13.5 ± 0.4^f^	–26.5 ± 0.3^f^
T49V/T78V/K79G	–137 ± 4	92 ± 3	na^h^	na^h^	–9.0 ± 1.0	13.2 ± 0.4	–24.4 ± 1.2

a Collected at 21 ± 1 °C.

b Calculated using relationships from Figure 1C.

c The midpoint reduction potential determined from averaging the reductive and oxidative spectroelectrochemical titrations.

d Calculated using relationships from Figure 1C, (–*nFE*_M‑M_^°′^ + *RT *ln *K*_eq_^III^)/–*nF*.

e Calculated using relationships from Figure 1C, (*–nFE*_K‑K_^°′^ – *RT* ln *K*_eq_^II^)/–*nF*.

f Reported in ref 35.

g K79G does not undergo Met-to-Lys ligand-switching at pH 7.4, and thus a cross-potential *nFE*_K‑M_°′ cannot be determined.

h T49V/T78V/K79G does not have all the necessary parameters using the relationships in Figure 1C to calculate this potential, and thus we determined this value experimentally.

**Table 6:** Thermodynamic Parameters for Ferric Variants of Yeast *iso*-1 Cyt *c* from GuHCl Unfolding Measurements at pH 4.5

variant	axial ligand^a^	*m*_D^III^_(kJ mol^–1^ M^–1^)^b^	Δ*G*_u_^III^°′(kJ mol^–1^)^b^
K79G^c^	Met80 (100%)	16.2 ± 3.1	17.7 ± 3.4
T49V/K79G^c^	Met80 (90–94%), H_2_O (6–10%)	11.4 ± 2.2	9.9 ± 2.2
T78V/K79G^c^	H_2_O (72–80%), Met80 (20–28%)	15.2 ± 3.2	13.2 ± 3.0
T49V/T78V/K79G	H_2_O (100%)	13.4 ± 3.9	11.8 ± 3.7

a Calculated using the corresponding p*K*_a_^III^ value from the pH titration and used for relative populations (*P*) of the H_2_O–Fe­(III)|TH^+^ and Met-Fe­(III)|TH^+^ states; ranges provided are determined from the error bars in the p*K*_a_^III^ values.

b Experimentally determined from GuHCl unfolding measurements (Figure S8A).

c Reported in ref 35.

### Energy Diagrams

The calculation of energy levels was done as described in the SI Methods. Figure 8 depicts the relative stability of the Met- and Lys-ligated species for ferric and ferrous T49V/T78V/K79G, as well as the other variants in this work at pH 7.4. Figure 9 compares the free energies of the ferric H_2_O-, Met-, and Lys-ligated states at pH 4.5. While the free energies of the H_2_O-ligated states of K79G and T49V/K79G are increased relative to the free energies of their Met-ligated states, the free energies of the H_2_O-ligated states for T49V/T78V/K79G and T78V/K79G are decreased with respect to those of their Met-ligated counterparts. The differences in trends suggest that incorporating Val mutations near the heme can provide a source of either stabilization or destabilization of cyt *c*. While disruption of the hydrogen-bonding network to an HP is expected to be unfavorable, incorporation of a hydrophobic residue in this region may provide an opportunity for new, favorable hydrophobic contacts to be formed. For instance, our modeling suggests that the Thr-to-Val substitution at residue 78 results in a clash between the Val side chain and a carbon on HP6 (Figure S13A,B). Thus, an alternative conformation is likely adopted by T78V/K79G, and perhaps by T49V/T78V/K79G.

**Figure 8:**
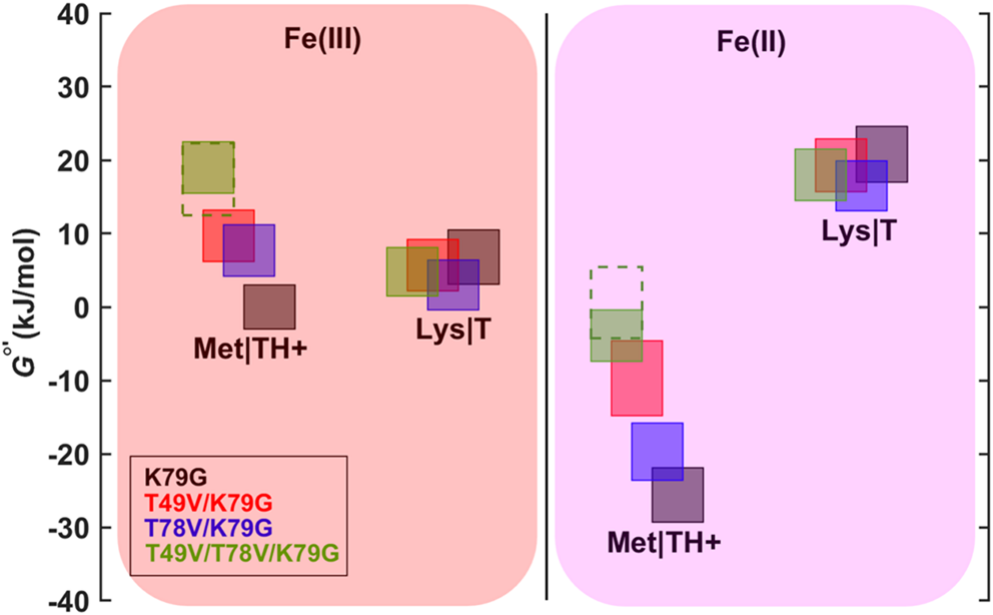
Energy diagram for the alkaline transition in ferric and ferrous variants at pH 7.4. Rectangles outlined with dashed lines are the free energy values for T49V/T78V/K79G obtained from the additive combination of the changes in the free energy values relative to those of K79G for the corresponding states of T49V/K79G and T78V/K79G. Heights of rectangles represent error bounds. The *x*-axis is used to separate the states of the alkaline transition in this diagram and does not have a quantitative meaning.

**Figure 9:**
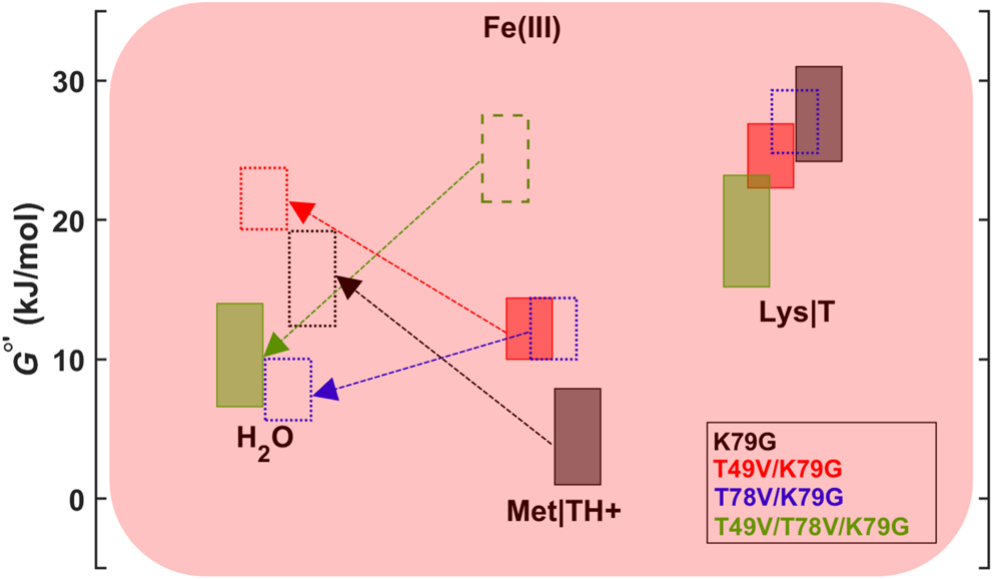
Free energies of differently ligated ferric variants at pH 4.5 from calculations and GuHCl denaturation experiments (solid rectangles) as detailed in the SI Methods (rectangles outlined with dotted lines). The rectangle outlined with dashed lines is the free energy value for T49V/T78V/K79G obtained from the additive combination of the changes in the free energy values for the corresponding states of T49V/K79G and T78V/K79G relative to K79G. Heights of the rectangles represent error bounds. The *x*-axis is used to separate the states of the alkaline transition in this diagram and does not have a quantitative meaning.

## Discussion

### Hydrogen Bonds to HPs Affect Cyt *c* Stability and Ligand Identity at the Heme Iron

Protein stability is a result of a delicate balance of a multitude of interactions. Metal–ligand interactions add to this collective action. The Fe–S bond in the absence of protein constraints is fairly weak (∼25 and 11 kJ mol^–1^ for Fe­(III) and Fe­(II), respectively);^32,33^ the structure of the surrounding polypeptide strengthens this bond in cyt *c*. The protein entatic contribution for ferrous horse heart cyt *c* was determined to be ∼17 kJ mol^–1^ from X-ray spectroscopy measurements.^33^ A similar estimate came from unfolding studies of this protein in the presence of CO.^69^

Modifications of the intraprotein hydrogen-bonding network affect the stability, as well as local and global conformational properties of cyt *c*.^19,31,34–36,38,70^ Consistent with their involvement in the control of Met ligation, NMR signals of amides of Asn52, Tyr67, and Thr78 are affected by Met80-to-CO ligand substitution.^69^ However, several adjacent residues within the larger hydrogen-bonding network are also perturbed, including Thr49. As we illustrate in this study, altering connections of residues 49 and 78 affects protein stability and ligation at the heme iron, suggesting that changes at the protein periphery propagate to the metal site. This finding adds to the developing understanding of the connection between the protein surface and the heme iron.^71,72^

### Mutations Progressively Destabilize Met-Ligated Cyt *c* to Abolish This Ligation in Ferric T49V/T78V/K79G

Steady-state spectroscopic studies have found no evidence of Met-ligated ferric T49V/T78V/K79G within the wide pH range studied. As our analyses show (Figure 8), each Thr-to-Val mutation adds to the destabilization of the Met-ligated state, and within error bars, the two substitutions combined destabilize the ferric and ferrous proteins by similar amounts (19.0 ± 3.5 and 23.1 ± 4.8 kJ mol^–1^, respectively). This effect is sufficient to remove the entatic imposition of Met80 coordination to the heme iron in the ferric protein, but not the more stable ferrous protein.

None of the mutations in the series we present here affect the secondary structure of the ferric or ferrous proteins (Figure S1A,B). Our prior studies have attributed destabilization effects in T49V/K79G and T78V/K79G primarily to local changes at the periphery of the heme, near HP6.^35^ A tight polypeptide fold and a similar heme solvent-accessible surface area to that in K79G were found for the Met-ligated forms of these two variants, in both the ferric and ferrous states. Near-UV CD (Figure S1D) and Trp59 fluorescence spectra (Figure S2B) for the Met-ligated ferrous T49V/T78V/K79G suggest some changes in the tertiary structure compared to those in T49V/K79G and T78V/K79G; however, the differences are minor. Since Met-ligated ferric T49V/T78V/K79G is not observable, its protein fold cannot be directly assessed, but the Trp59 spectra of the H_2_O-ligated ferric protein (Figure S5) suggest greater changes in the overall compactness of the protein ensemble when both Thr-to-Val mutations are present.

The foldon formalism and its extensive applications to horse heart cyt *c* offer insights as to why the ferric protein is more perturbed by the two Thr-to-Val mutations than the ferrous protein and why the latter retains its Met80 ligation.^14,15^ Ferrous cyt *c* is more stable than its ferric counterpart,^73–75^ and the stabilization upon the heme iron reduction has been observed for each of its foldons (Figure S14A). The stability of cyt *c* depends on the oxidation state of the iron in part because the formal charge of +1 of the ferric heme group is destabilizing for the folded protein and the two oxidation states differ in solvation.^73^ While the information on the free energies of foldons for yeast *iso*-1 cyt *c* is limited, the framework is applicable to other cytochromes and, more broadly, to other proteins.^15^ Studies by Bowler et al. suggest that the free-energy range of foldons in the less stable yeast cyt *c* is more compressed than that in the horse heart cyt *c* (Figure S14A,B).^41^ The free energy associated with global unfolding has often been used to estimate the free energy of the most stable blue foldon in cyt *c*.^14,41^ Taking into account the decrease in the stability of the Met-ligated T49V/T78V/K79G compared to that of K79G (Figure 8), we may expect the blue foldon in the former variant to be destabilized. With the dramatic shift in the level of the blue foldon in T49V/T78V/K79G, below the free energies of the lower-lying foldons in the wild-type protein (Figure 10), the stabilities of the lower-lying foldons in this variant are also expected to be lower.

**Figure 10:**
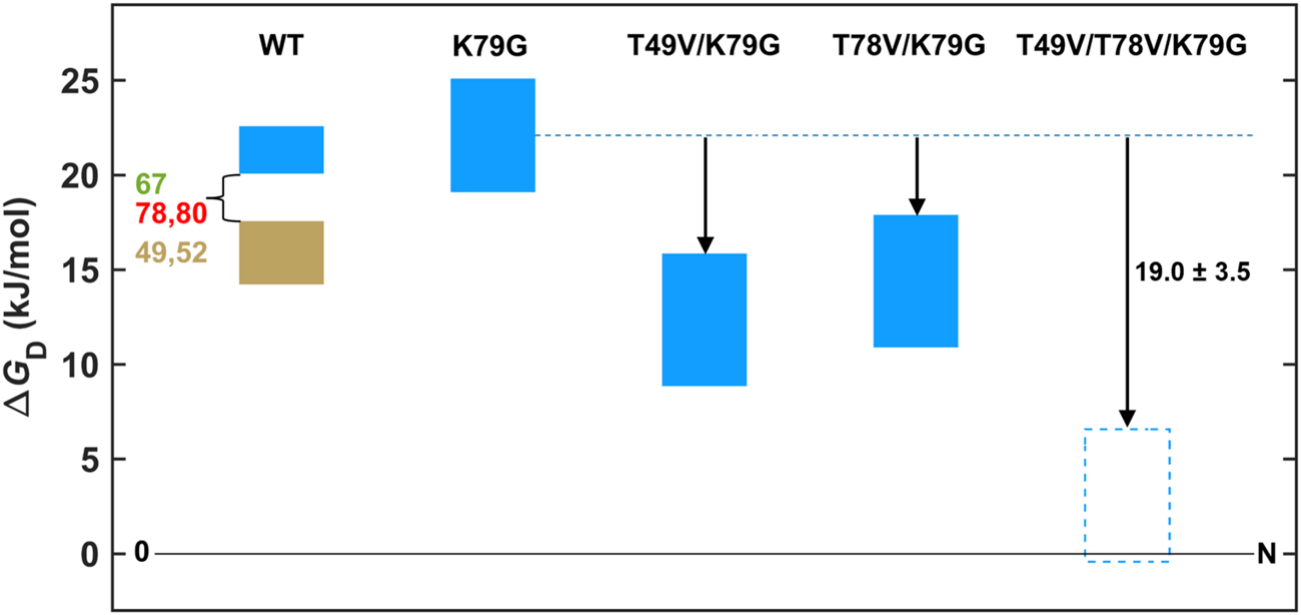
Stabilities of the foldons in ferric WT yeast *iso*-1 cyt *c* from limited proteolysis (infrared (tan)) and GuHCl denaturation (blue) experiments, plotted based on the tabulated data in ref 41. The free energies of the red and green foldons were not determined in yeast *iso*-1 cyt *c*. The solid (K79G, T49V/K79G, and T78V/K79G) and dashed-outlined (T49V/T78V/K79G) rectangles depict the free energies of the most stable blue foldons in the ferric Met-ligated proteins; these coincide with the values in Figure 8. Heights of rectangles represent error bounds.

Among the three residues in the entatic control region, Tyr67 is particularly noteworthy as it belongs to a more stable green foldon (Figure 1A). While some mutations at this site preserve the Met ligation,^76,77^ others greatly affect the structure of the protein, abolishing the Met80 ligation.^34,77,78^ The lack of the Met-ligated species for ferric T49V/T78V/K79G suggests that the red and possibly green foldons are likely unfolded in this protein, consistent with the known placement of the foldons (Figure S14) and the destabilization energy we determined for T49V/T78V/K79G (Figure 8). For ferric T49V/K79G and T78V/K79G, the stability of the blue foldon (judged by the effects on the global stability) is less perturbed, which may not be sufficient to shift the lower-lying foldons to the range when they are unfolded (Figure 10). For ferrous T49V/T78V/K79G, the low-lying foldons are more stable and do not unfold,^14,17^ which preserves the Met ligation.

### Mutations Have a Minor Effect on the Stability of Lys-Ligated Cyt *c*

In the structure of the Lys73-ligated yeast *iso*-1 cyt *c* variant with an altered heme coordination loop, Thr49 no longer hydrogen bonds to HP6 through its side chain, which is exposed, but instead through the backbone amide nitrogen.^38^ Further, residue 78 is not within hydrogen-bonding distance for interactions with HP6 in this and other models of the Lys73-ligated protein.^38,79,80^ While the Lys-ligated protein is not expected to experience a penalty from the removal of the hydrogen-bonding connections upon the mutations, it may experience destabilization of the unfolded state from substituting a Thr with a hydrophobic Val.^81^ However, this effect is likely minor as the stabilities of the three Lys-ligated Thr-to-Val variants are all within error of that of K79G (Figure 8).^82^

### Properties of the H_2_O-Ligated Protein

The calculated energy levels (Figures 8 and 9) reveal two interesting features of the H_2_O-ligated T49V/T78V/K79G. First, in contrast to estimates for the Met- and Lys-ligated forms of this variant, it appears that the effects of the two Thr-to-Val mutations are not additive for the H_2_O-ligated protein. Second, in contrast to the H_2_O-ligated states of K79G and T49V/K79G that are destabilized with respect to their Met-ligated counterparts, the H_2_O-ligated states of T49V/T78V/K79G and T78V/K79G are stabilized. Indeed, spectroscopic data (Figure S3A,B) suggest that at pH 4.5 ferric T49V/T78V/K79G is exclusively H_2_O-ligated and the broader signals in the downfield region of the NMR spectrum of T49V/T78V/K79G at pD 4.5 (Figure S4A) hint to a conformational exchange in this protein, consistent with a more open heme pocket.

The stability of the H_2_O-ligated M80A at pH 5.0 (p*K*_a_ = 5.9 ± 0.2 for the H_2_O-to-OH^–^ transition) is lower than that of the Met-ligated wild-type cyt *c*.^47^ Similarly, K79G, which still has Met80, is destabilized upon the Met-to-H_2_O transition (Figure 9). Several structures of cyt *c* variants are available to probe whether perturbation in hydrogen bonds that HP6 forms with Thr49 and Thr78 may be relevant in such destabilization. A yeast *iso*-1 cyt *c* variant with a K72A mutation exhibited H_2_O ligation at the heme iron with Met80 no longer in the heme pocket, but HP6 still remained within hydrogen-bonding distance to both Thr49 and Thr78.^23^ An NMR structure of cyanide-bound M80A cyt *c* has revealed a similar protein fold to that of the wild-type protein.^82^ The Thr49-HP6 hydrogen bond is retained in the structure of this adduct, and the Thr78-HP6 connection is also present, but only in a small fraction of the ensemble. In the structure of Im-bound horse heart cyt *c*, neither Thr49 nor Thr78 form hydrogen bonds with HP6.^83^ These examples illustrate that the loss of Met ligation does not necessitate the loss of the hydrogen-bonding interactions of HP6 with the two Thr, but larger perturbations (such as those upon Im coordination) do. As Met coordination does add to the protein stability,^73^ one would expect the stability of the H_2_O-ligated proteins to decrease relative to their Met-ligated counterparts, and this is the effect observed for K79G and T49V/K79G (Figure 9).

The findings that stabilities of the H_2_O-ligated T78V/K79G and T49V/T78V/K79G increase relative to their Met-ligated counterparts suggest that in these variants, the unfavorable interactions from Met80 in the heme pocket (Figure S13) are somehow relieved. One explanation is that the expulsion of Met80 from the heme pocket may result in favorable interactions. There may be some parallels to the structure of a lipid-bound structure of cyt *c*,^26^ in which Thr78 and Met80 swing out from the heme pocket, and Asn52, Lys55, Val57, Tyr74, and Ile75 cluster to form a hydrophobic pocket. It is possible that in T78V/K79G and T49V/T78V/K79G, where Thr78 is replaced by Val, Val78 and Met80 form hydrophobic interactions. For T49V/T78V/K79G, where the degree of stabilization upon loss of the Met80 ligation is greater and there are observable perturbations in the tertiary structure (Figure S2), new favorable interactions may involve Val49 as well as other residues.

The stabilization of H_2_O-ligated T49V/T78V/K79G alone, however, does not explain why the Met-ligated state is not observed in this variant. Relative to their counterparts for K79G, the H_2_O-ligated form of T49V/T78V/K79G at pH 4.5 is stabilized by only 5.5 ± 5.0 kJ mol^–1^, while the Met-ligated form of T49V/T78V/K79G is apparently destabilized by 20.0 ± 4.6 kJ mol^–1^ (Figure 9). A similar destabilization (19.0 ± 3.5 kJ mol^–1^) is observed at pH 7.4, in comparing the Met-ligated forms of K79G and T49V/T78V/K79G. With this extent of destabilization, the Met-ligated form of the ferric protein is no longer populated at equilibrium, and as our preliminary kinetics studies suggest (Figure S11), it also rapidly disappears in the transient experiments. The increased polypeptide dynamics upon destabilization of multiple low-lying foldons is likely the cause of the kinetic effect.

### Protein Destabilization as a Way to Engineer Redox-Linked Ligand Switching

Redox-linked ligand switching is a useful attribute to control charge separation and ET directionality. Acting on the coordinated ligand itself, through its protonation or substitution by an exogenous ligand, is one way to introduce such a function. Acting on the surrounding frame is another way to change the coordinated ligand in a particular oxidation state. We have previously described a series of cyt *c* variants where a thiolate (a strong ligand for ferric hemes) was introduced in the heme iron vicinity, resulting in thiolate-ligated ferric proteins and differently ligated ferrous proteins.^67^

As this report shows, destabilizing the metalloprotein by modifying more distant elements of the protein structure is another way to change the coordinating ligand in a redox-dependent manner. Because of the sequential nature of cyt *c* unfolding,^14,17^ destabilizing the low-energy foldons also destabilizes the more stable foldons critical for the protein scaffold (and entatic control). With global destabilization by ∼20 kJ mol^–1^, the heme iron ligation of Met is disabled in ferric but not ferrous cyt *c*, suggesting the relative placement of the red and green foldons in these forms of the protein (Figure S14). While the value of the destabilization coincides with the estimate of the entatic contribution in horse heart cyt *c*,^32^ simply destabilizing the protein by this amount does not result in loss of the Met ligation, as seen with ferrous T49V/T78V/K79G. As long as the protein scaffold is maintained, the perturbation will remain localized. Further, the entatic contribution in the less stable yeast cyt *c*, which has not been determined yet, may be lower.

The case of cyt *c* is not unique, and other metal and ligand combinations could be imagined. The stabilities of the oxidized and reduced states of metalloproteins differ,^73,84^ with one or the other being more stable depending on the metal, ligand, or the protein. As a consequence, destabilization of low-lying foldons in the less stable state could remove the entatic control of a particular ligation. With a suitable alternative endogenous ligand nearby, a redox-linked switchable function is created.

## Conclusions

The mutations of two Thr residues in the periphery of the heme additively destabilize Met-ligated cyt *c*, disabling this ligation in the ferric protein at equilibrium. A H_2_O-ligated state forms instead, and thermodynamic analyses of this series of mutants have revealed the energetic properties of this state and offered insights into the possible formation of new favorable contacts. The destabilization of the entatic state in ferric cyt *c* was quantified, demonstrating that its control extends beyond the metal site to peripheral interactions. Destabilization of the scaffold of redox metalloproteins can be employed as a mechanism to engineer differently ligated oxidation states of particular interest in the design of switchable systems for numerous applications.^85–87^

## Supplementary Material



## Notes

Additional methods describing energy diagram calculations for ferric and ferrous K79G, T49V/K79G, T78V/K79G, and T49V/T78V/K79G at pH 7.4; energy diagram calculations for ferric K79G, T49V/K79G, T78V/K79G, and T49V/T78V/K79G at pH 4.5; tables showing cross-potentials of T49V/K79G, T78V/K79G, and T49V/T78V/K79G from spectroelectrochemistry; parameters for the alkaline transition of ferric K79G, T49V/K79G, T78V/K79G, and T49V/T78V/K79G; figures showing CD spectra of ferric and ferrous K79G, T49V/K79G, T78V/K79G, and T49V/T78V/K79G at pH 7.4; fluorescence spectra of ferric and ferrous K79G, T49V/K79G, T78V/K79G, and T49V/T78V/K79G at pH 7.4; corresponding electronic absorption spectra and the SVD analysis for the pH titration data of ferric T49V/T78V/K79G; ^1^H NMR spectrum of ferric T49V/T78V/K79G, ferric horse heart M80A cyt *c*, and ferric yeast *iso*-1 K79G cyt *c* at pD 4.5; fluorescence spectra of ferric and ferrous K79G, T49V/K79G, T78V/K79G, and T49V/T78V/K79G at pH 4.5; absorbance changes at 415 nm for the ferrous pH titration of T49V/T78V/K79G; NMR and CD spectroscopic changes for the pH titration of ferrous T49V/T78V/K79G; cyclic voltammograms at 0.1 V/s and 1 V/s of T49V/T78V/K79G at pH 7.4; cyclic voltammograms of T49V/T78V/K79G at pH 7.4 and 0.1 V/s varying the lower potential limit; cyclic voltammograms of T49V/T78V/K79G at pH 6.0 and pH 5.0; cyclic voltammogram of T49V/T78V/K79G at pH 7.4 and 10 V/s; digital simulation of voltammetry of T49V/T78V/K79G at 10 V/s; spectral changes during stopped-flow mixing of ferrous T49V/T78V/K79G with K_3_Fe­(CN)_6_; proposed heme iron ligation states during stopped-flow mixing of ferrous T49V/T78V/K79G with K_3_Fe­(CN)_6_; GuHCl titrations of ferric K79G, T49V/K79G, T78V/K79G, and T49V/T78V/K79G at pH 7.4 and 4.5; models of yeast *iso*-1 cyt *c* showing possible clashes between a heme propionate and Val substituted at residue 78 in Chimera; energies of foldons in reduced and oxidized horse heart cyt *c*; and energies of foldons in reduced and oxidized yeast *iso*-1 cyt *c* (PDF)
