# Supplementary material for: Disabling the Entatic Control of Methionine Ligation through Additive Destabilization of Ferric Cytochrome c
Source: Inorg Chem. 2025 Jun 11;64(24):11966–80. doi: 10.1021/acs.inorgchem.5c00839 (PMC12188557; doi:10.1021/acs.inorgchem.5c00839)
Supplement: Supplementary file 1 [file ic5c00839_si_001.pdf]

Disabling the entatic control of methionine ligation through additive destabilization of ferric cytochrome *c*

Morgan E. Reik[a], Taylor C. Rickett[b], Kevin R. Hoke[b], Ekaterina V. Pletneva[a*]

[a]Department of Chemistry, Dartmouth College, Hanover, New Hampshire 03755, United States

[b]Department of Chemistry and Biochemistry, Berry College, Mount Berry, Georgia 30149, United States

*Corresponding author: ekaterina.pletneva@dartmouth.edu

Supporting Information

SI Methods

Energy diagram of ferric and ferrous variants at pH 7.4. The $\Delta G_u^{\circ\prime III}$ values from GuHCl measurements of ferric variants allowed for placement of the Lys-Fe(III)|T energy levels relative to those of the Met-Fe(III)|TH^+ species.[1] For T49V/K79G and T78V/K79G, the spectroscopically determined $pK_a{}^{III}{}_{M\text{-}K}$ values were used to calculate a pH-adjusted ligand exchange equilibrium constant $K_{eq}{}^{III}$ to find the free energies of the Met-Fe(III)|TH^+ species according to $\Delta G_{K\text{-}Fe(III)}{}^{\circ\prime} - \Delta G_{M\text{-}Fe(III)}{}^{\circ\prime} = -RT\ln K_{eq}{}^{III}$, where $K_{eq}{}^{III} = K_a{}^{III}/[H^+]$.[1] The rate constant $k_{off}{}^{Lys}$, together with our estimate of $k_f{}^{III}$, was used to calculate the equilibrium constant $K_a{}^{III}{}_{M\text{-}K}$ and the free energy change $\Delta G_{M\text{-}K}{}^{\circ\prime III}$ for the alkaline transition in ferric T49V/T78V/K79G, and ultimately $\Delta G_{M\text{-}Fe(III)}{}^{\circ\prime}$ of the Met-ligated ferric species although this species has not been explicitly observed. Similar calculations for the other variants reproduce $\Delta G_{M\text{-}Fe(III)}{}^{\circ\prime}$ values from $pK_a{}^{III}{}_{M\text{-}K}$ values (**Table 5**).

Experimental $E_{K\text{-}K}{}^{\circ\prime}$ (from direct electrochemistry) and $E_{K\text{-}M}{}^{\circ\prime}$ (from spectroelectrochemistry) values were employed to calculate $pK_{eq}{}^{II}$ values (**Fig. 1C**) for T49V/T78V/K79G, from which $pK_a{}^{II}{}_{M\text{-}K}$ values were determined (**Table 2**). The energy levels for Lys-Fe(II)|T in each variant were found using $E_{K\text{-}K}{}^{\circ\prime}$, where $\Delta G_{K\text{-}Fe(II)}{}^{\circ\prime} = \Delta G_{K\text{-}Fe(III)}{}^{\circ\prime} - nFE_{K\text{-}K}{}^{\circ\prime}$. The value of $pK_{eq}{}^{II}$ for T49V/T78V/K79G from spectroelectrochemistry was used to estimate the energy of the Met-Fe(II)|TH^+ species according to the relationship $\Delta G_{K\text{-}Fe(II)}{}^{\circ\prime} - \Delta G_{M\text{-}Fe(II)}{}^{\circ\prime} = -RT\ln K_{eq}{}^{II}$.

Energy diagram of ferric variants at pH 4.5. Since at pH 4.5 ferric T49V/T78V/K79G is H_2O-ligated (**Fig. 3A** and **Table 6**), the free energy of its H_2O-Fe(III)|TH^+ species was quantified using $\Delta G_u{}^{\circ\prime}(H_2O)^{III}$ from denaturation experiments. With this value, $pK_a{}^{III}{}_{H_2O\text{-}K}$ was used to place the Lys-ligated state of this variant on the energy diagram by calculating the pH-adjusted ligand

exchange equilibrium constant K_{eq}^{III}, as has been done at pH 7.4.[1] Similarly, since both K79G and T49V/K79G are Met-ligated at pH 4.5 (**Table 6**), results from denaturation experiments were used to find the free energy of their Met-Fe(III)|TH$^+$ states, and the positions of the Lys-ligated states for these variants was obtained from the corresponding p$K_a^{III}{}_{M\text{-}K}$ values. With the knowledge of relative populations (P) of the H_2O-Fe(III)|TH$^+$ and Met-Fe(III)|TH$^+$ states (K79G and T49V/K79G)[1,2] and H_2O-Fe(III)|TH$^+$ and Lys-Fe(III)|T states (T49V/T78V/K79G, **Fig. 3A**) from pH titration experiments, the free energy of the H_2O-ligated states was calculated using $\Delta G_{H_2O\text{-}Fe(III)}^{\circ\prime} = \Delta G_{M\text{-}Fe(III)}^{\circ\prime} - RT\ln(P_{H_2O\text{-}Fe(III)}/P_{M\text{-}Fe(III)})$.

At pH 4.5, T78V/K79G exists in a mixture of H_2O- and Met-ligated species (**Table 6**).[1] We assumed that the free energies of the Met-ligated T49V/K79G and T78V/K79G are similar at pH 4.5, as they are within error at pH 7.4.[1] The $\Delta G_u^{\circ\prime}(H_2O)^{III}$ of T49V/K79G, which is >90% Met-ligated at pH 4.5,[3] was used as the free energy value representing a fully Met-ligated case. The free energy of the H_2O-Fe(III)|TH$^+$ state of T78V/K79G was then calculated from the populations of the H_2O-Fe(III)|TH$^+$ and Met-Fe(III)|TH$^+$ states as above and the position of its Lys-Fe(III)|T state was calculated from the corresponding p$K_a^{III}{}_{M\text{-}K}$ value.

Table S1. Cross-potentials of variants of yeast *iso*-1 cyt *c* from spectroelectrochemistry determined from measurements performed in oxidative and reductive directions[a]

Variant	$E_{K\text{-}M}$°′ (mV)		
	$Fe^{II} \rightarrow Fe^{III}$	$Fe^{III} \rightarrow Fe^{II}$	Averaged
T49V/K79G	163 ± 1	131 ± 6	147 ± 3
T78V/K79G	211 ± 1	193 ± 2	202 ± 1
T49V/T78V/K79G	106 ± 3	78 ± 4	92 ± 3

[a]Data and corresponding fits are shown in **Fig. 6**

Table S2. Parameters pK_C, pK_H, and pK_a for the alkaline transition in ferric variants of yeast *iso*-1 cyt *c*

**Variant**	**pK_C^{IIIa}**	**pK_H^{III}**	**pK_a^{IIIb}**
K79G[c]	−1.9 ± 0.2	10.5 ± 0.1	8.6 ± 0.2
T49V/K79G[c]	−1.5 ± 0.1	8.2 ± 0.2	6.7 ± 0.2
T78V/K79G[c]	−1.8 ± 0.2	8.4 ± 0.2	6.6 ± 0.3
T49V/T78V/K79G	−3.5 ± 0.1	8.3 ± 0.2[d]	4.9 ± 0.2

[a] $K_c^{III} = k_f^{III}/k_b^{III}$

[b] pK_a^{III} = pK_C^{III} + pK_H^{III}

[c] Reported in ref.[1]

[d] As the deprotonation process cannot be experimentally observed for T49V/T78V/K79G, pK_H^{III} for this variant is reported as the average of that of T49V/K79G and T78V/K79G. Since the effect of mutations on pK_H^{III} is particularly strong for T49V/K79G and T78V/K79G compared to other variants of cyt *c*,[4–6] we assume that pK_H^{III} in T49V/T78V/K79G is comparable.

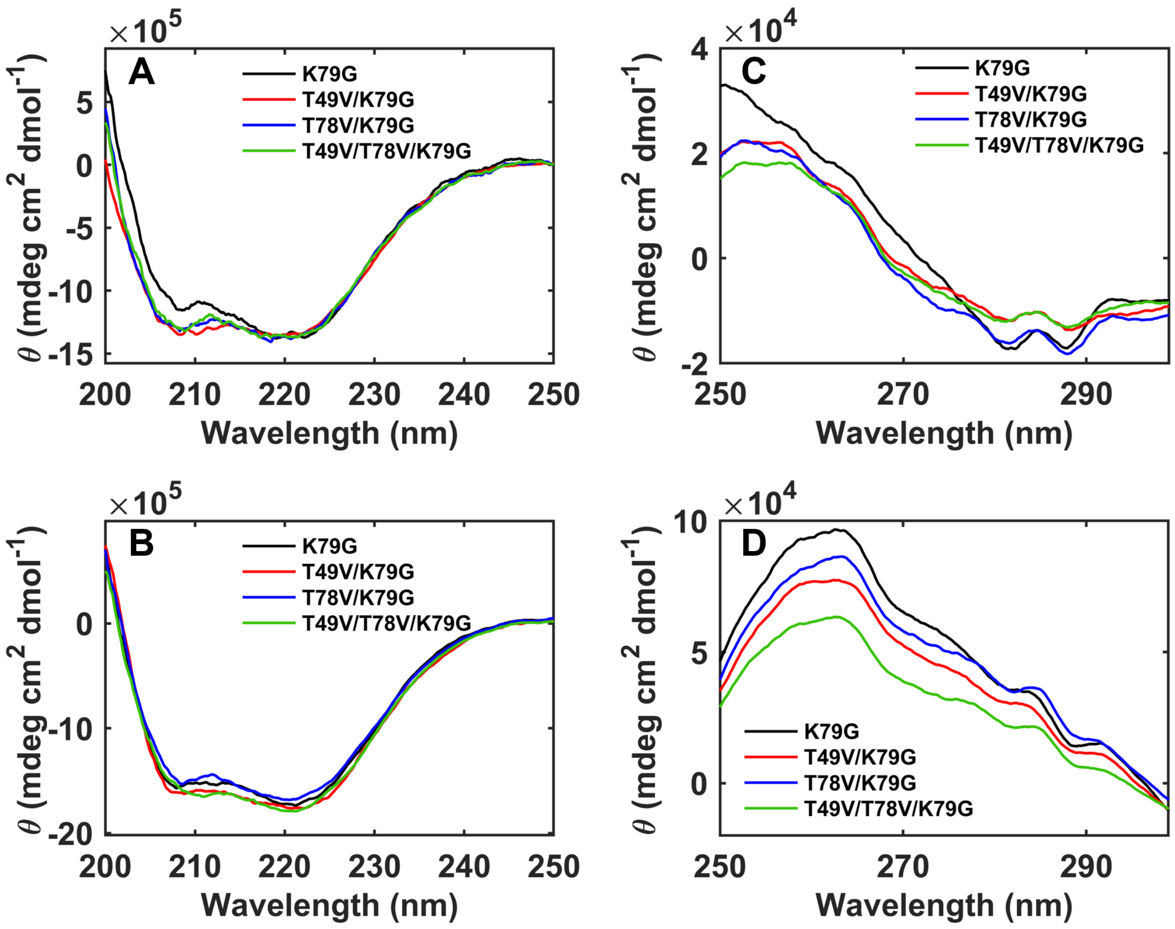

Figure S1. Far-UV (A, B) and near-UV (C, D) CD spectra of ferric (A, C) and ferrous (B, D) variants at pH 7.4.

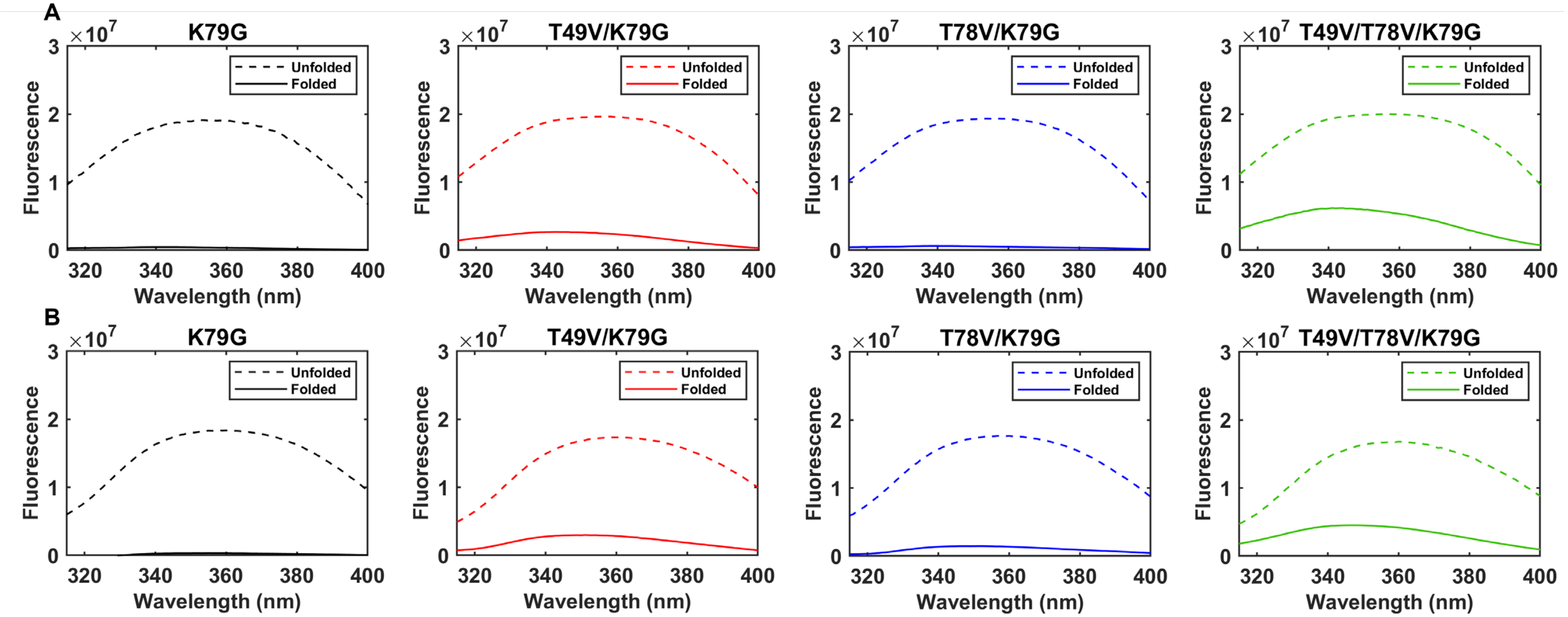

Figure S2. Fluorescence spectra (λ_{ex} = 290 nm) of (A) ferric and (B) ferrous variants in a 100 mM sodium phosphate buffer at pH 7.4 (folded) and in 5 M GuHCl at pH 7.4 (unfolded). Protein concentrations were 10 µM.

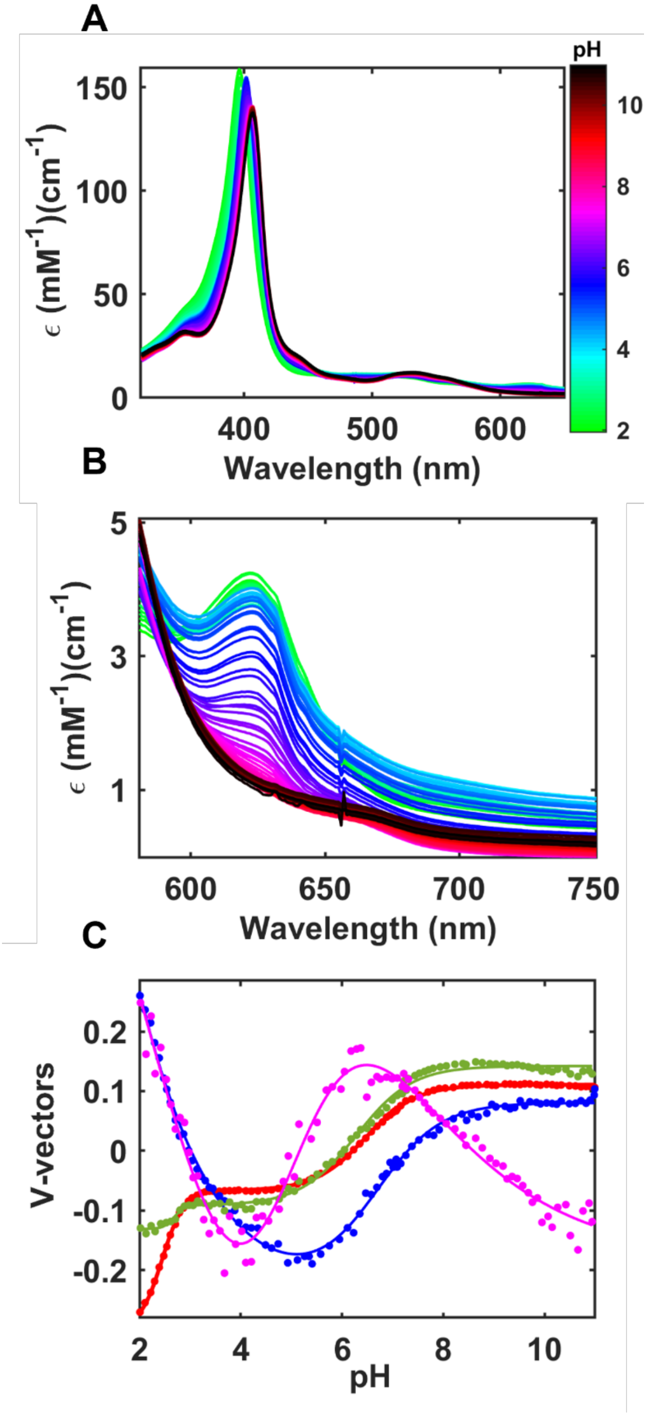

Figure S3. Electronic absorption spectra of ferric T49V/T78V/K79G as a function of pH: (A) the Soret band and (B) the charge-transfer band regions. (C) Global fitting of the most weighted V-vectors to **Eq. 1** from two pH titrations. The V-vectors corresponding to the titration monitoring the Soret region are depicted in red and blue, and the V-vectors corresponding to the titration monitoring the charge-transfer region are depicted in pink and green.

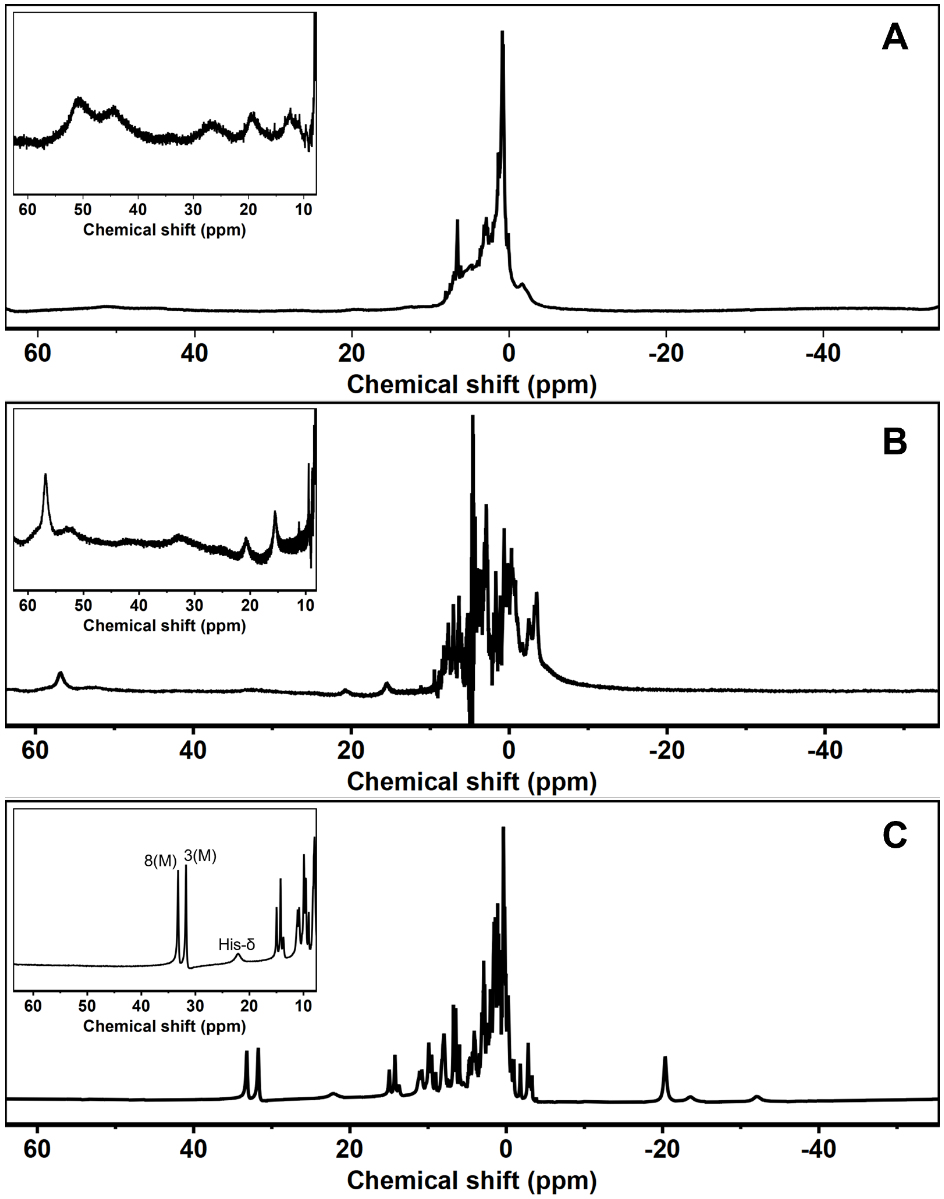

Figure S4. ^{1}H NMR spectra at pD 4.5 of (A) T49V/T78V/K79G *iso*-1 cyt *c*, (B) M80A horse heart cyt *c*, and (C) K79G *iso*-1 cyt *c*. Insets depict the downfield region of the NMR spectrum.

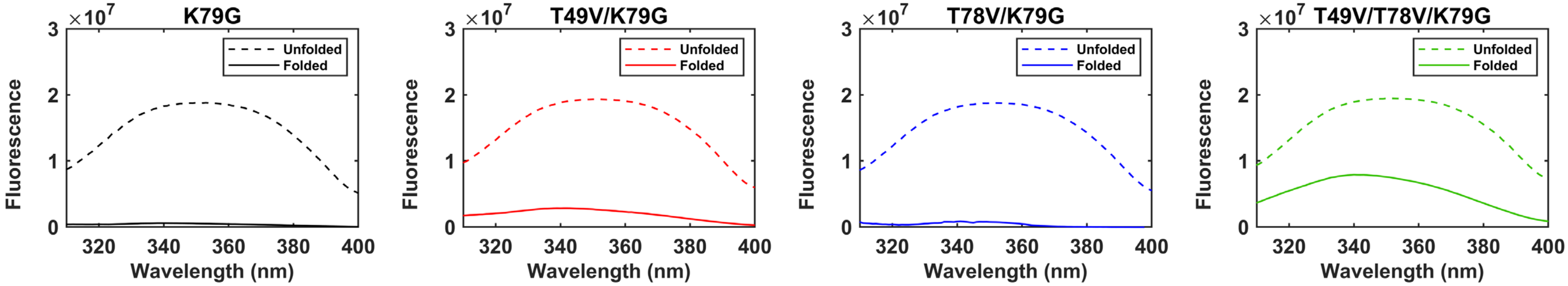

Figure S5. Fluorescence spectra (λ_{ex} = 290 nm) of ferric variants in a 100 mM acetate buffer at pH 4.5 (folded) and 5 M GuHCl at pH 4.5 (unfolded). Protein concentrations were 10 μM.

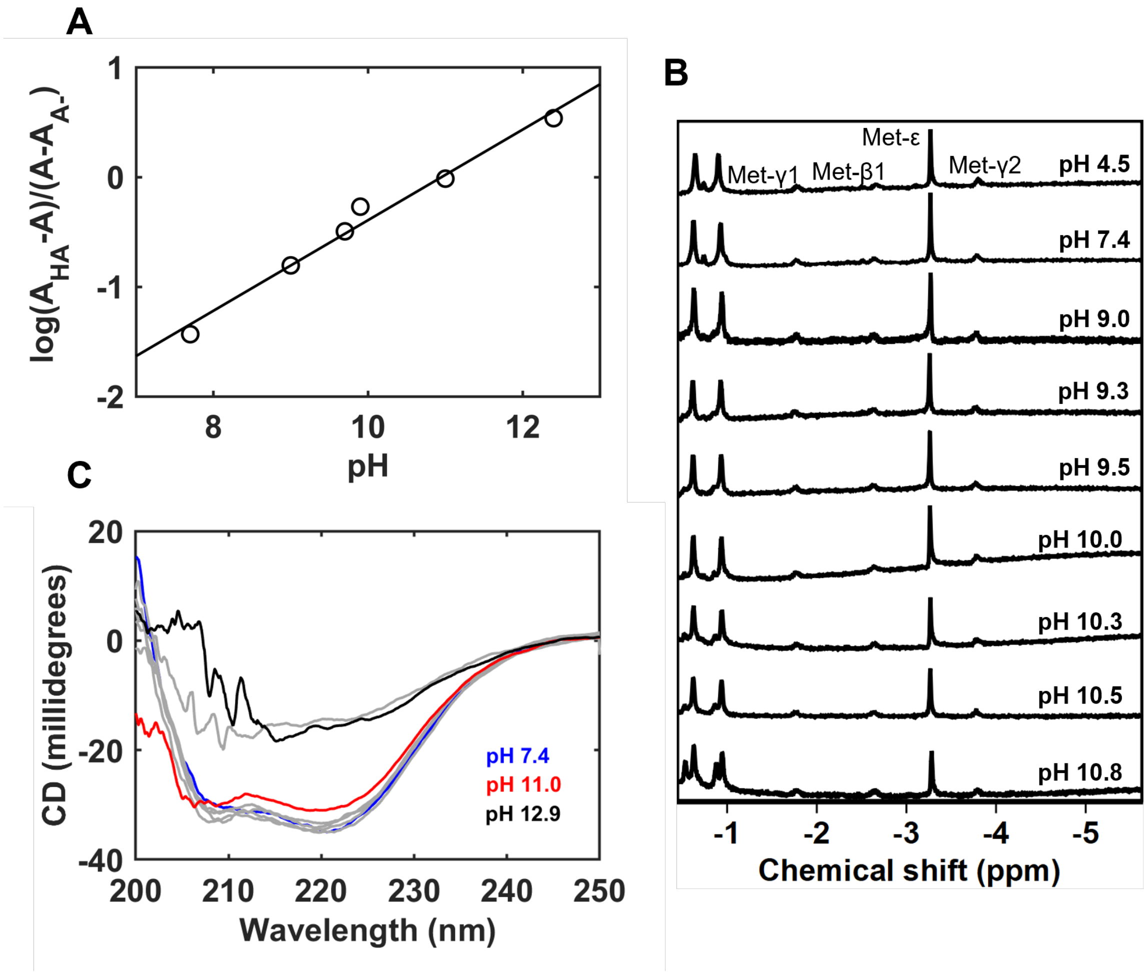

Figure S6. (A) The absorbance changes at 415 nm (λ_{max} of the Soret band) of ferrous T49V/T78V/K79G with pH. The pK_a value was found using the *y*-intercept of the semi-log plot according to the Henderson-Hasselbalch equation. (B) NMR pH titration of ferrous T49V/T78V/K79G monitoring the Met80 protons. (C) CD spectra of ferrous T49V/T78V/K79G in the far-UV region monitoring changes in the secondary structure with pH. The spectra at pH 7.4, 11.0, and 12.9 are shown in blue, red, and black, respectively; the spectra at other pH values monitored are in gray.

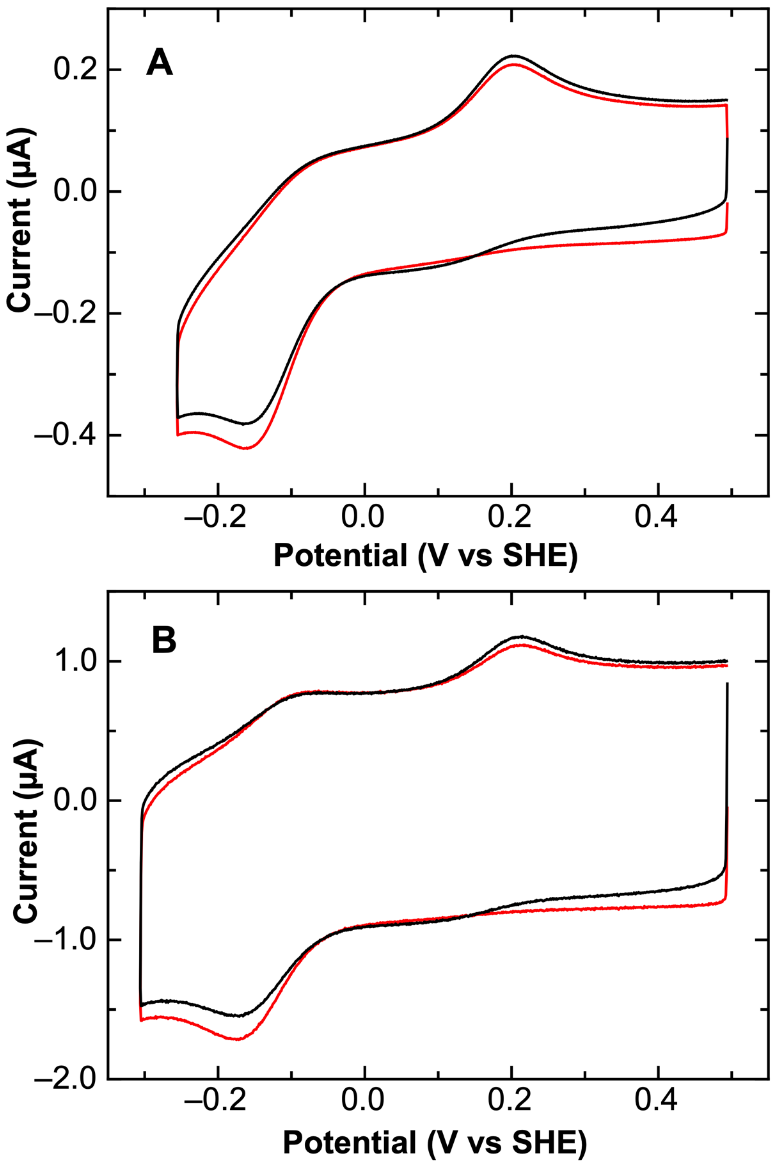

Figure S7. CV for T49V/T78V/K79G at pH 7.4 and (A) 0.1V/s and (B) 1 V/s. The first cycle of two is depicted in red, the second in black. The protein concentration was 135 μM.

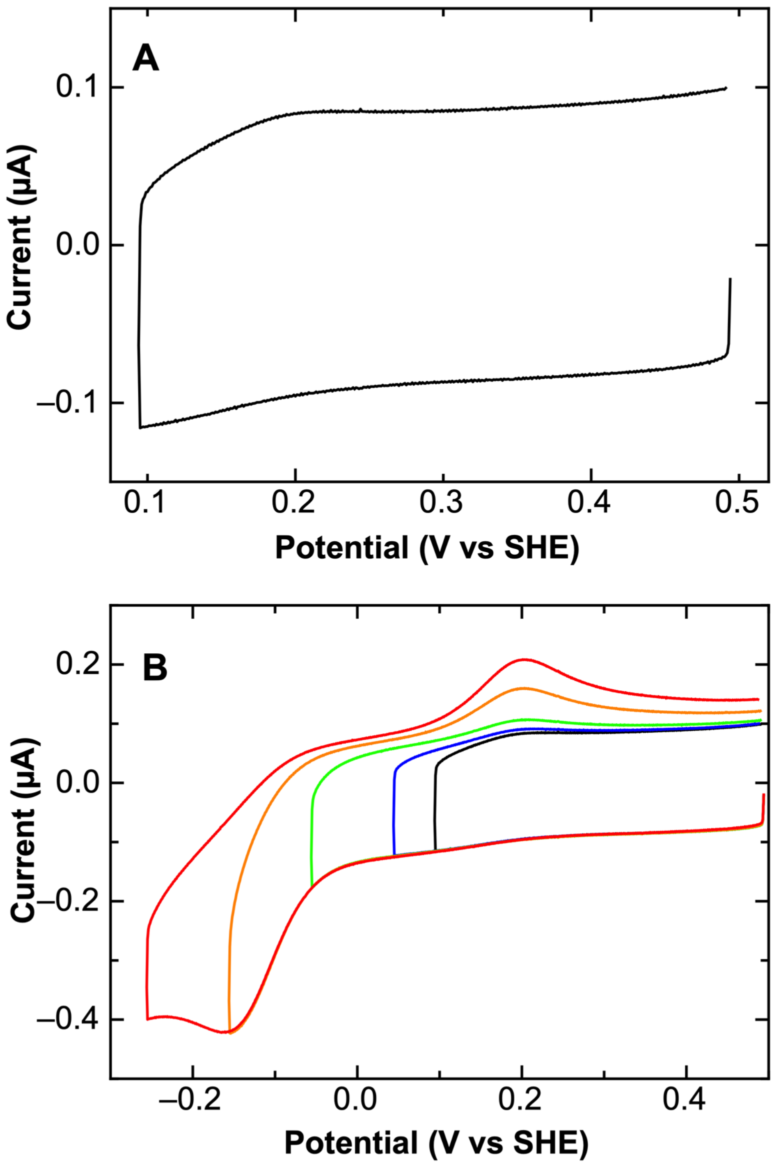

Figure S8. CV for T49V/T78V/K79G at pH 7.4 and 0.1 V/s, starting from upper potential limit and showing only first cycles. (A) Voltammogram over a relatively high potential range. (B) Overlay of multiple voltammograms acquired in quick succession showing the effect of progressively decreasing the lower potential limit. The protein concentration was 135 μM.

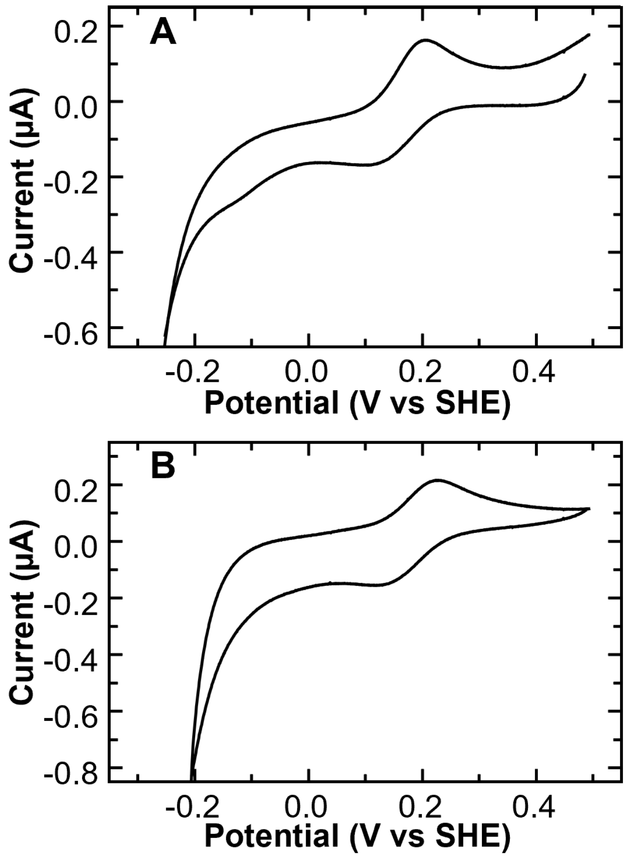

Figure S9. CV for T49V/T78V/K79G at 0.1 V/s with background current subtracted at (A) pH 6.0 and (B) pH 5.0. The second cycle of two is shown for each. The protein concentration was 120 μM.

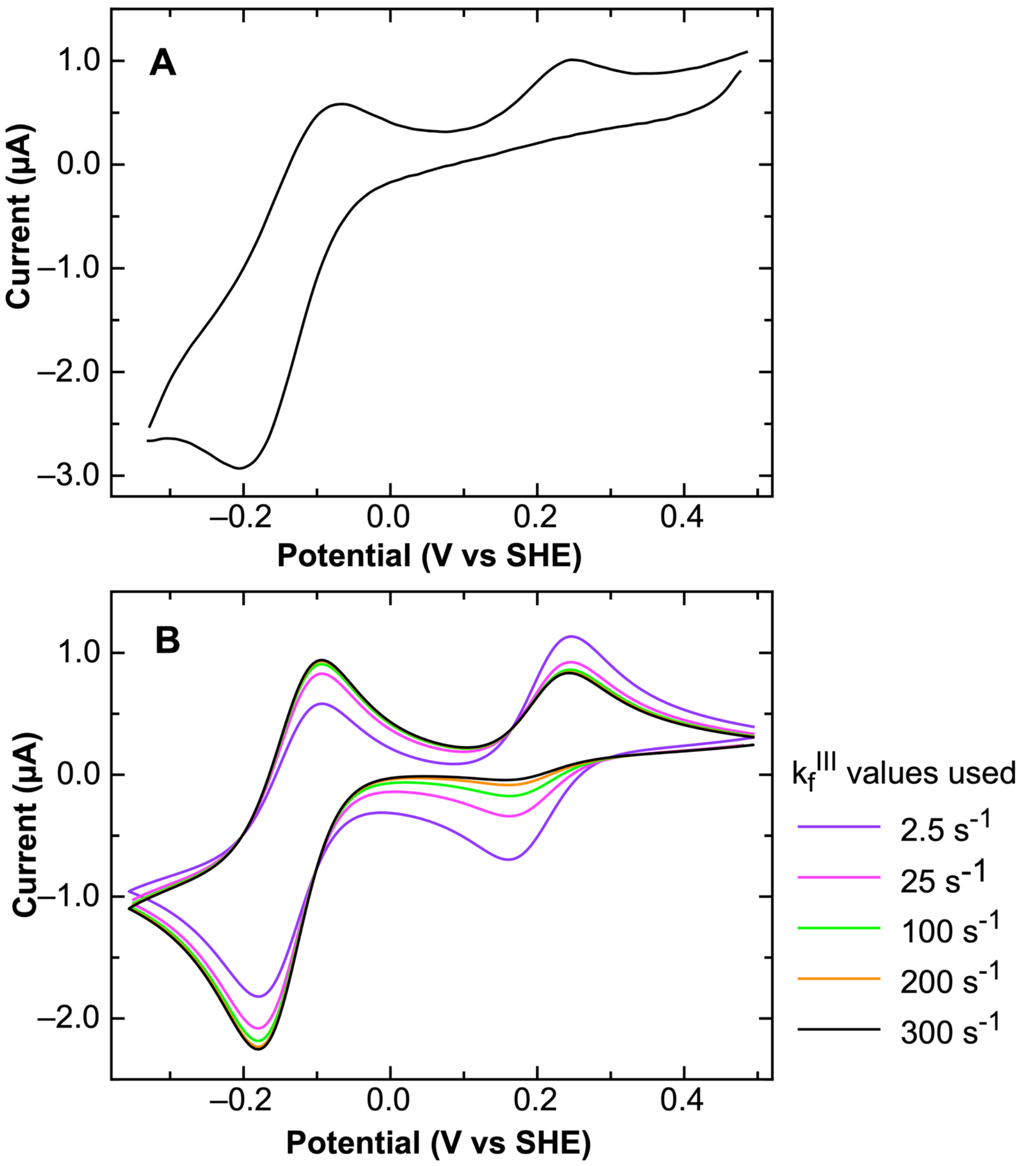

Figure S10. (A) CV for T49V/T78V/K79G at pH 7.4 and 10 V/s, showing second cycle with background current subtracted and noise reduction by Fourier filtering. Protein concentration was 135 µM. (B) Digital simulation of the voltammetry at 10 V/s. The value of k_f^{III} was varied in the simulation and the resulting peaks compared to observed voltammetry. Only the second full cycles are shown for clarity, but both cycles were simulated and used in the analysis. Other parameters in simulations include E_{low} = -140 mV, E_{high} = +200 mV, k_b^{III} = 0.113 s^{-1}, k_b^{II} = 16 s^{-1}, heterogenous electron transfer rate, k_o = 0.025 cm/s, and diffusivity constant, D = 8.0 × 10^{-7} cm^2/s. The latter two values are from ref.[7] Since only one higher potential signal is clear in the data, potentials associated with a H_2O-ligated species and a possible Met-ligated species are not separable in the simulation.

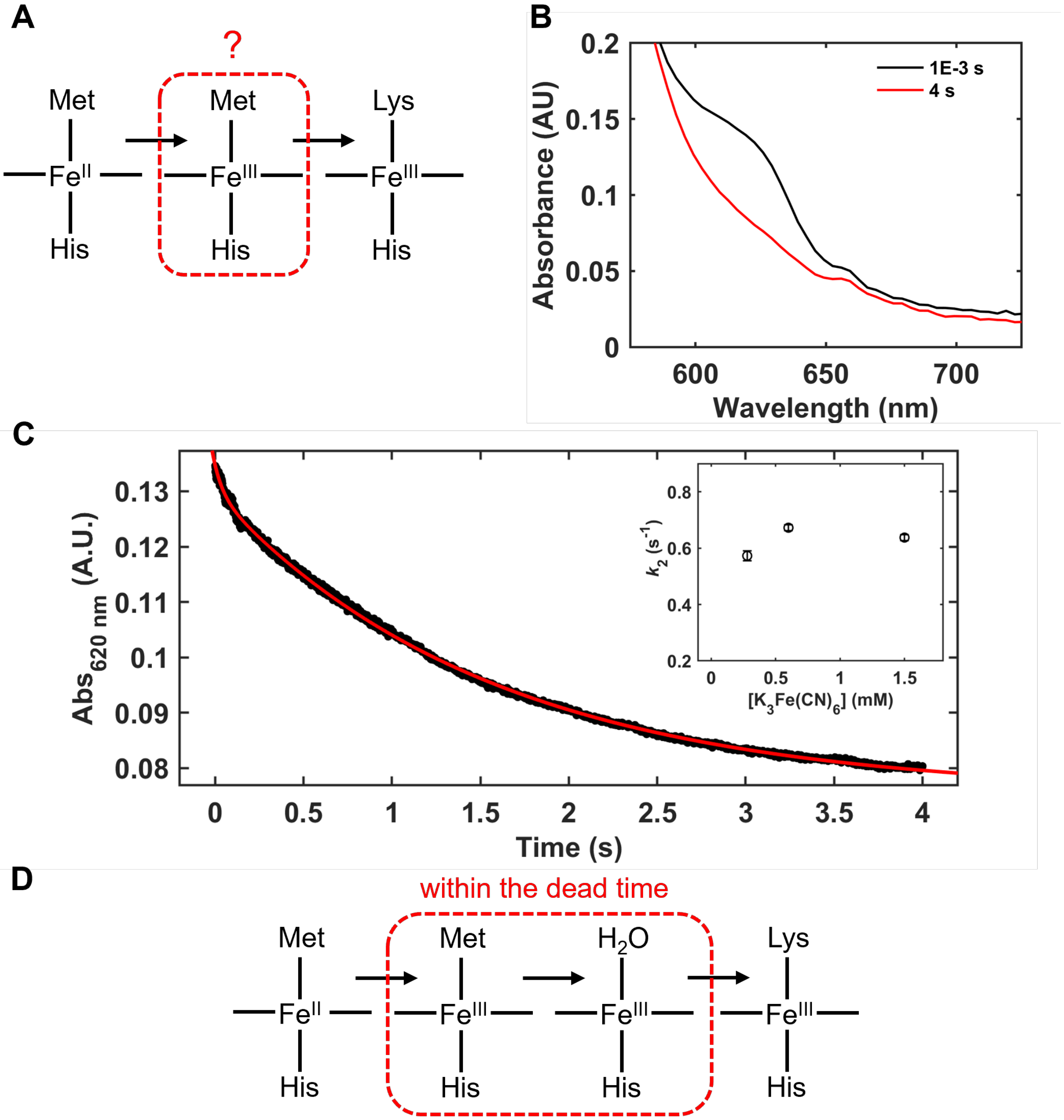

Figure S11. Oxidation of ferrous T49V/T78V/K79G by $K_3Fe(CN)_6$ at pH 7.4. (A) This process was expected to yield a transient Met-ligated ferric species as in our prior work with other variants.[8,9] (B) Electronic absorption spectra at 1 ms (black) and 4 s (red) after stopped-flow mixing. Concentrations of T49V/T78V/K79G and $K_3Fe(CN)_6$ were 50 µM and 1.5 mM, respectively. (C) Spectral changes at 620 nm with time and corresponding fit to a biexponential decay function yielding k_1 (9%) = 19.7 ± 1.6 s^{-1} and k_2 (91%) = 0.64 ± 0.004 s^{-1}; inset depicts k_2 values at different concentrations of $K_3Fe(CN)_6$. (D) States considered to be present during the experiment.

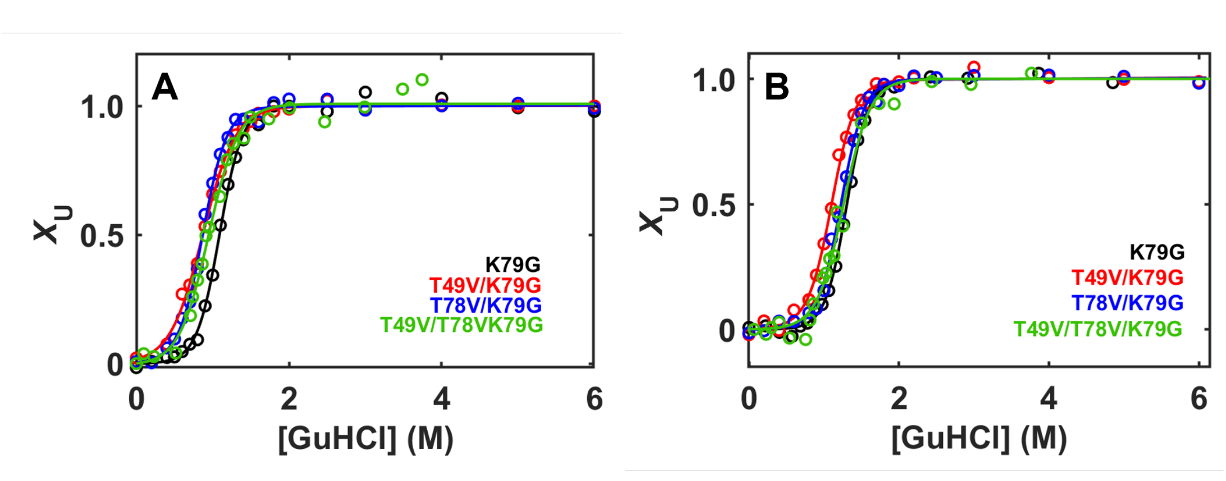

Figure S12. Results of GuHCl unfolding of ferric variants monitored by CD spectroscopy at (A) pH 4.5 and (B) pH 7.4; curves were fit to **Eq. 6**.

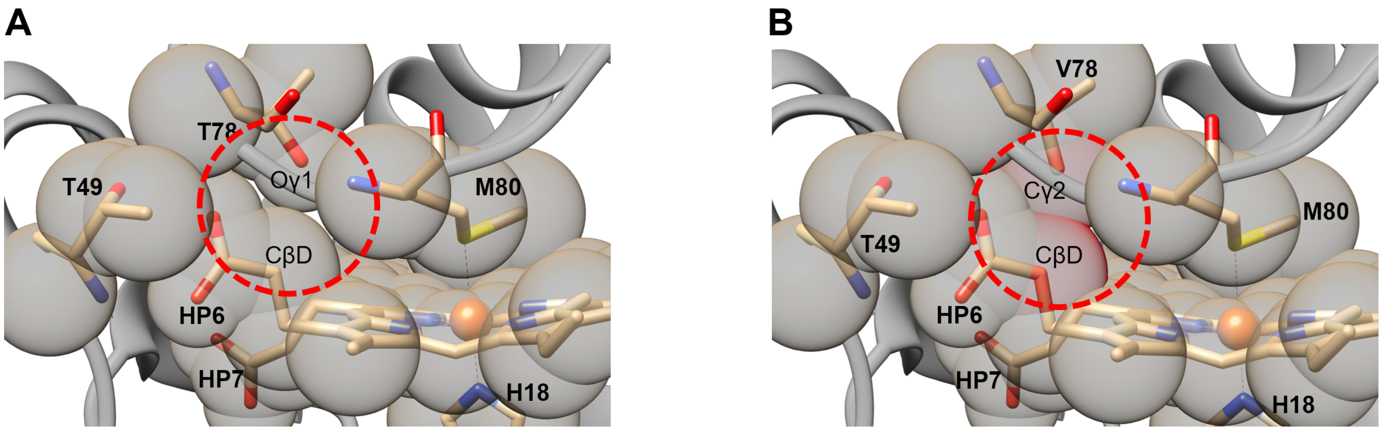

Figure S13. (A) Contacts involving studied residues from crystal structure of yeast *iso*-1 cyt *c* (PDB ID: 2YCC)[10] and (B) the same image but with Val78 substituted for Thr78 in Chimera.[11] Val78 clashes with HP6 upon the substitution.

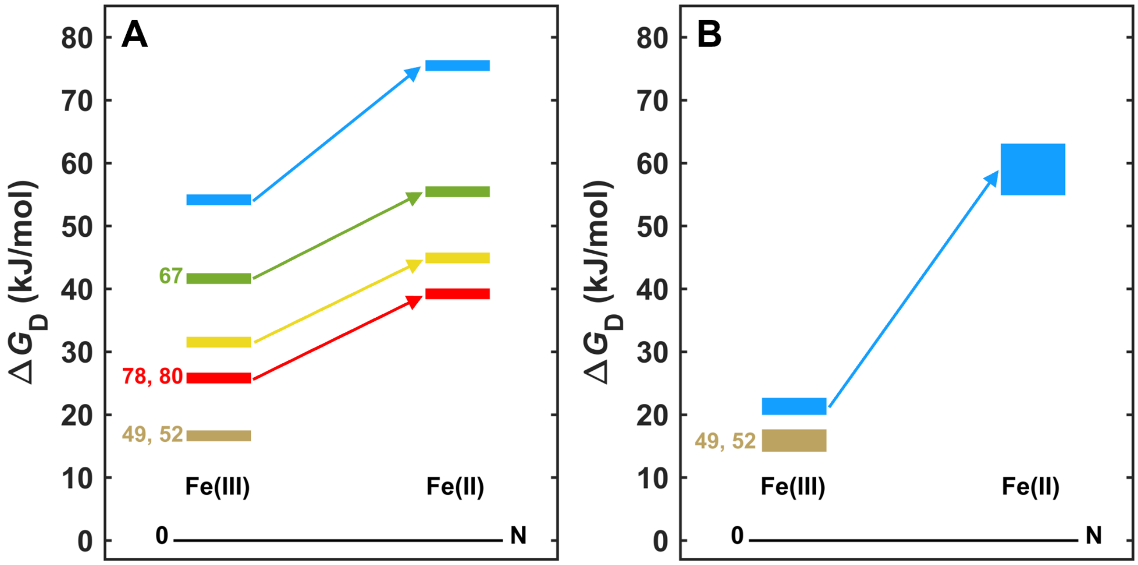

Figure S14. (A) ΔG_D of each foldon in ferric and ferrous horse heart cyt *c* from hydrogen-deuterium exchange at pD_r 7 (adapted with permission from ref.[12], copyright 2006, Elsevier). (B) Known ΔG_D values of foldons in ferric and ferrous yeast *iso*-1 cyt *c*.[13–15] Heights of rectangles in this figure represent error bounds.

SI References

(1) Deng, Y.; Weaver, M. L.; Hoke, K. R.; Pletneva, E. V. A Heme Propionate Staples the Structure of Cytochrome *c* for Methionine Ligation to the Heme Iron. *Inorg. Chem.* **2019**, *58* (20), 14085–14106.

(2) Amacher, J. F.; Zhong, F.; Lisi, G. P.; Zhu, M. Q.; Alden, S. L.; Hoke, K. R.; Madden, D. R.; Pletneva, E. V. A Compact Structure of Cytochrome *c* Trapped in a Lysine-Ligated State: Loop Refolding and Functional Implications of a Conformational Switch. *J. Am. Chem. Soc.* **2015**, *137* (26), 8435–8449.

(3) Deng, Y.; Zhong, F.; Alden, S. L.; Hoke, K. R.; Pletneva, E. V. The K79G Mutation Reshapes the Heme Crevice and Alters Redox Properties of Cytochrome *c*. *Biochemistry* **2018**, *57* (40), 5827–5840.

(4) Pearce, L. L.; Gartner, A. L.; Smith, M.; Mauk, A. G. Mutation-Induced Perturbation of the Cytochrome *c* Alkaline Transition. *Biochemistry* **1989**, *28* (8), 3152–3156.

(5) Deacon, O. M.; Svistunenko, D. A.; Moore, G. R.; Wilson, M. T.; Worrall, J. A. R. Naturally Occurring Disease-Related Mutations in the 40-57 Ω-Loop of Human Cytochrome *c* Control Triggering of the Alkaline Isomerization. *Biochemistry* **2018**, *57* (29), 4276–4288.

(6) Lei, H.; Nold, S. M.; Motta, L. J.; Bowler, B. E. Effect of V83G and I81A Substitutions to Human Cytochrome *c* on Acid Unfolding and Peroxidase Activity below a Neutral pH. *Biochemistry* **2019**, *58* (26), 2921–2933.

(7) Khoshtariya, D. E.; Dolidze, T. D.; Seifert, S.; Sarauli, D.; Lee, G.; Van Eldik, R. Kinetic, Thermodynamic, and Mechanistic Patterns for Free (Unbound) Cytochrome *c* at Au/SAM Junctions: Impact of Electronic Coupling, Hydrostatic Pressure, and Stabilizing/Denaturing Additives. *Chem. – Eur. J.* **2006**, *12* (27), 7041–7056.

(8) Zhong, F.; Pletneva, E. V. Mechanistic Studies of Proton-Coupled Electron Transfer in a Calorimetry Cell. *J. Am. Chem. Soc.* **2019**, *141* (25), 9773–9777.

(9) Zhong, F.; Alden, S. L.; Hughes, R. P.; Pletneva, E. V. Comparing Properties of Common Bioinorganic Ligands with Switchable Variants of Cytochrome *c*. *Inorg. Chem.* **2022**, *61* (3), 1207–1227.

(10) Berghuis, A. M.; Brayer, G. D. Oxidation State-Dependent Conformational Changes in Cytochrome *c*. *J. Mol. Biol.* **1992**, *223* (4), 959–976.

(11) Petterson, E. F.; Goddard, T. D.; Huang, C. C.; Couch, G. S.; Greenblatt, D. M.; Meng, E. C.; Ferrin, T. E. UCSF Chimera--a Visualization System for Exploratory Research and Analysis. *J. Comput. Chem.* **2004**, *25* (13), 1605–1612.

(12) Krishna, M. M.; Maity, H.; Rumbley, J. N.; Lin, Y.; Englander, S. W. Order of Steps in the Cytochrome *c* Folding Pathway: Evidence for a Sequential Stabilization Mechanism. *J. Mol. Biol.* **2006**, *359* (5), 1410–1419.

(13) Duncan, M. G.; Williams, M. D.; Bowler, B. E. Compressing the Free Energy Range of Substructure Stabilities in *Iso*-1-Cytochrome *c*. *Protein Sci.* **2009**, *18* (6), 1155–1164.

(14) Godbole, S.; Bowler, B. E. Effect of pH on Formation of a Nativelike Intermediate on the Unfolding Pathway of a Lys 73 → His Variant of Yeast *Iso*-1-Cytochrome *c*. *Biochemistry* **1999**, *38* (1), 487–495.

(15) Mines, G. A.; Winkler, J. R.; Gray, H. B. Spectroscopic Studies of Ferrocytochrome *c* Folding. *ACS Symp. Ser.* **1998**, *692*, 198–211.
